# Cotton under heat stress: a comprehensive review of molecular breeding, genomics, and multi-omics strategies

**DOI:** 10.3389/fgene.2025.1553406

**Published:** 2025-03-18

**Authors:** Tahira Luqman, Manzoor Hussain, Syed Riaz Ahmed, Iram Ijaz, Zahra Maryum, Sahar Nadeem, Zafran Khan, Sana Muhy Ud Din Khan, Mohammad Aslam, Yongming Liu, Muhammad Kashif Riaz Khan

**Affiliations:** ^1^ Nuclear Institute for Agriculture and Biology-Constituent College (NIAB-C), Pakistan Institute of Engineering and Applied Science Nilore, Islamabad, Pakistan; ^2^ Plant Breeding and Genetics Division, Cotton Group, Nuclear Institute for Agriculture and Biology, Faisalabad, Pakistan; ^3^ Horticulture Research Institute, Pakistan Agriculture Research Council (PARC), Khuzdar, Pakistan; ^4^ Department of Botany, University of Agriculture Faisalabad, Faisalabad, Pakistan; ^5^ Department Plant Breeding and Genetics, University of Agriculture Faisalabad, Faisalabad, Pakistan; ^6^ National Nanfan Research Institute (Sanya), Chinese Academy of Agricultural Sciences, Sanya, China,

**Keywords:** cotton, heat stress, molecular breeding, genome editing, multi-omics

## Abstract

Cotton is a vital fiber crop for the global textile industry, but rising temperatures due to climate change threaten its growth, fiber quality and yields. Heat stress disrupts key physiological and biochemical processes, affecting carbohydrate metabolism, hormone signaling, calcium and gene regulation and expression. This review article explores cotton’s defense mechanism against heat stress, including epigenetic regulations and transgenic approaches, with a focus on genome editing tools. Given the limitations of traditional breeding, advanced omics technologies such as GWAS, transcriptomics, proteomics, ionomics, metabolomics, phenomics and CRISPR-Cas9 offer promising solutions for developing heat-resistant cotton varieties. This review highlights the need for innovative strategies to ensure sustainable cotton production under climate change.

## 1 Introduction

Cotton (*Gossypium hirsutum*) is one of the most important cash crops, providing natural fiber to textile industries worldwide and supporting the livelihoods of over a hundred million households (Zhang Z. et al., 2023). Annually, cotton contributes approximately $600 billion to the global economy (Tokel et al., 2022). Beyond fiber, cotton serves as a food source; nearly 65% of conventional cotton products enter the food chain, either directly through cottonseed oil or indirectly through meat and milk from animals consuming cottonseed meal and ginning by-products (Saini et al., 2023; Wang W. et al., 2023; Zia et al., 2021).

Globally, cotton is cultivated in nearly 35 countries, covering approximately 34.1 million hectares and yielding about 120 million bales annually (Shi et al., 2023; AOF, 2023). China, India, and the United States collectively contribute around 60% of total cotton production (Meyer and Dew, 2021). China, being the largest cotton consumer, utilizes around 7.60 million tons yearly (Zhu et al., 2023). The United States, being the third largest cotton producer and top exporter, holds an important position in the global cotton market (USDA ERS, 2020a).

However, cotton production faces continuous threats from climate change, particularly heat stress (USDA ERS, 2020b). Elevated temperatures adversely affect cotton’s growth, development and yield. For example, in the southwestern United States, heat stress has led to a 26% reduction in cotton yields (Elias et al., 2018; Riisgaard et al., 2020). In Arizona’s low desert, cottonseed yields are projected to reduce by 40% by the mid-century (2036–2065) and by 51% by the late-century (2066–2095), compared to the baseline period of 1980–2005 (Ayankojo et al., 2020).

The rising concentrations of greenhouse gases (GHGs) and rapid environmental changes pose severe challenges to the sustainability of agriculture (Voora et al., 2023). Global warming is expected to lead to more abrupt and extreme environmental fluctuations in agricultural regions (Gupta et al., 2020). For example, in Pakistan, the average annual temperature is predicted to rise by 4.38°C by 2080 (Tariq et al., 2023; Shehzad, 2023). As sessile organisms, plants are continuously exposed to various abiotic stress, including drought, salinity, heavy metals and heat stress, which affect their survival, growth, development and yield (Elahi et al., 2022). Among these, heat stress is one of the most serious threats to global food security, with reports indicating that each degree Celsius increase in temperature can reduce crop yields by over 17% during the growing season for major crops (Elahi et al., 2022; Riisgaard et al., 2020). Therefore, it is not difficult to predict the drastic effects of increased temperature on agricultural production (Dutta et al., 2023).

Countries like USA, China, India, Pakistan, Brazil, Turkey and Australia have the capacity to produce greater than the average cotton yield at slightly higher temperatures, accounting for about 75% of the world’s cotton production area (Tokel et al., 2022). However, if the average annual temperature continues to rise at the current rate, even these leading cotton-producing countries will face production losses (Meyer and Dew, 2021). Regions already producing cotton at around 40°C would suffer greatly from hostile climatic conditions, leading to dramatic losses in the production per unit area (Yousaf et al., 2023).

Given the critical role of cotton in global agriculture and its vulnerability to heat stress (He et al., 2024), it is imperative to explore strategies to mitigate the impact of rising temperatures. While only few previous studies (Saleem et al., 2021; Majeed et al., 2021; Abro et al., 2023) have explored the effects of heat stress on cotton, mostly have primarily focused on either physiological responses or traditional breeding approaches. This review advances current knowledge by providing a comprehensive synthesis of recent findings on the morphological, physiological, and biochemical responses of cotton to heat stress. Additionally, it explores cutting-edge omics technologies, including genomics, transcriptomics, proteomics, and metabolomics, to understand heat stress tolerance mechanisms at a molecular level. Unlike earlier reviews, this work integrates emerging strategies such as CRISPR-Cas gene editing, genome-wide association studies (GWAS), and high-throughput phenotyping to accelerate the development of heat-resilient cotton cultivars. By bridging conventional and modern breeding techniques, this review provides a multidisciplinary perspective on improving cotton’s adaptability to climate change. Ultimately, it aims to guide future research and breeding programs by identifying genetic and biotechnological interventions that can enhance cotton’s resilience, ensuring sustainable production in a warming climate.

## 2 Effect of heat stress on cotton morphology and physiology

**FIGURE 1:**
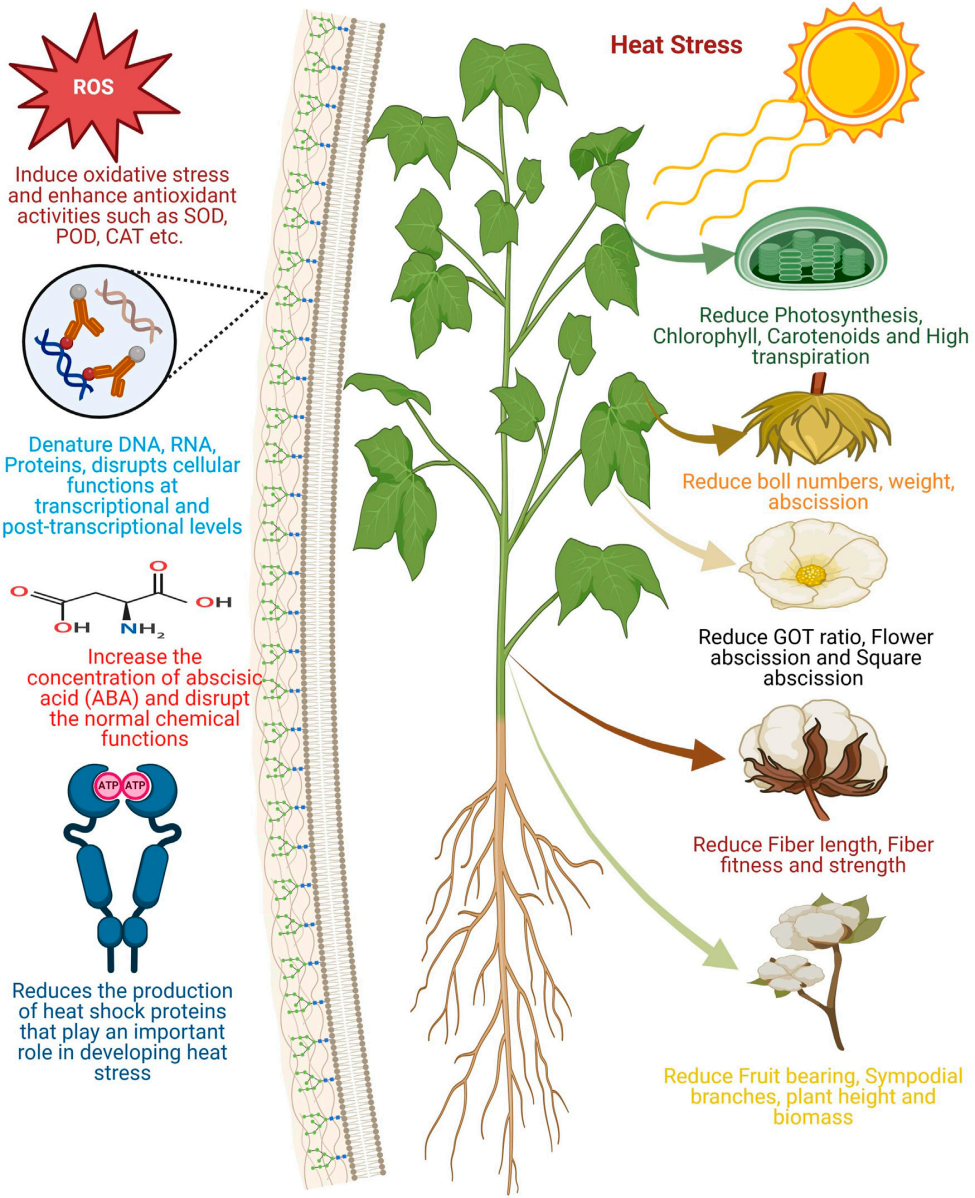
Illustration of the general effects of heat stress or elevated temperatures on cotton growth and development. Heat stress reduces the photosynthesis rate, chlorophyll, and carotenoid contents. It also decreases the number and weight of cotton bolls, ginning outturn (GOT) ratio, and increases flower and square abscission. Additionally, it shortens fiber length, weakens fiber strength, lowers fiber quality, and reduces fruit-bearing capacity and the number of sympodial branches. Heat stress leads to an increase in reactive oxygen species (ROS) concentration, accompanied by elevated levels of superoxide dismutase (SOD), peroxidase (POD), and catalase (CAT). At the molecular level, it causes the denaturation of DNA, RNA, and key cellular enzymes, thereby impairing the optimal production of heat shock proteins essential for heat stress mitigation. Note: this figure was generated using BioRender.

High temperature has a significant effect on cotton growth and reproduction. Heat stress adversely affects cotton growth throughout its life cycle, with the reproductive stages being more sensitive to high temperatures than other growth stages (Majeed et al., 2021; Ma et al., 2021). The optimal temperature for optimal cotton seedling growth is reported to be 30°C (Majeed et al., 2021) (although few studies have also reported a range of 30°C–34°C (Beegum et al., 2024; Sarwar et al., 2023; Mudassir et al., 2021). High temperature has a very crucial effect on different developmental stages of the cotton such as germination., seedlings growth, vegetative propagation, and traits of maturity and morphological development are very important (Sarwar et al., 2023) (Figure 1). Yousaf et al. (2023) studied the effects of heat stress on morphological traits of several upland cotton genotypes. Heat stress significantly effected all the studied traits, including plant height, nodes per plant, sympodial branches, bolls per plant, ginning out-turn, and staple length. Approximately 33%–46% of reduction was observed in these traits compared to the control, highlighting the detrimental impact of heat stress on cotton morphology. Similarly, Mudassir et al. (2021) evaluated the impact of varying high temperatures on cotton morphology across six major cotton-growing cities in Pakistan. Yield-related traits were severely affected at all locations, leading to significant yield losses. Boll size, number of bolls, and number of flowers were significantly reduced by approximately 47%–54% on average across all locations. The effect of heat stress on pollen tube germination, growth and elongation indicates that temperatures >30°C adversely affect cotton reproductive performance (Zhang et al., 2024). Pollen germination was maximum when the temperature was regulated at 28°C (Saud and Wang, 2022). The rate of germination was inverse to a temperature >28°C and it declined rapidly at temperatures >37°C (Saud and Wang, 2022). Zhang et al. (2024) reported that significant reduction in boll weight and the number of bolls per plant under heat stress was strongly associated to lower pollen fertility. This reduction in pollen fertility was further associated with disruptions in energy metabolism and anther carbohydrate balance. Heat stress enhanced sucrose content in anthers by limiting sucrose hydrolysis due to reduced activities of invertase and sucrose synthase. However, sucrose hydrolysis can be accelerated to mitigate pollen infertility under heat stress by downregulating the expression of the *GhSWEET55*, *GhSUT4*, and *GhSUT3A/D* genes.

### 2.1 Root growth

Roots are the first plant organs that get exposed to any abiotic stress before other plant parts (Farooq et al., 2024). Healthy plant growth mainly depends on the health of the roots, which is affected by many factors, with temperature being one of the most critical (Virk et al., 2021). For better growth, cotton should be cultivated during a specific period to minimize the negative effects of environmental stressors. The ideal temperature range for root development is 22°C–30°C during the day and 27°C–35°C at night (Reddy V. et al., 1997). However, temperature exceeding 32/40°C disrupt root growth, causing roots to become shorter and stunted roots even with abundant nutrients and water availability (Fan et al., 2022). Heat stress severely impacts root traits, leading to significant reductions in root length (34%–67%, depending on the severity of temperature) (Virk et al., 2021; Parkash et al., 2024; Xia et al., 2023; Saleem et al., 2021), and root total biomass (41%–77%) (Wang Q. et al., 2024; Dev et al., 2024; Wu et al., 2022), thereby meshing the overall root system architecture (Guo et al., 2024a). Heat stress also diminishes the development of primary root hairs (Yu et al., 2022; Fan et al., 2022), root elongation (Rani et al., 2022; Yousaf et al., 2023), branching (Fan et al., 2022), and ultimately inhibit the uptake of essential nutrients (Hannan et al., 2024; Saleem et al., 2021), and water required for cotton survival (Shifa et al., 2024; Das et al., 2024). Moreover, in arid and semi-arid regions, heat tolerance in seedlings is a key factor for successful plant cultivation (Naveed et al., 2025), because maintaining optimal soil moisture during sowing is essential to support healthy seedling growth (Ma et al., 2024). Increased wind velocity and high soil temperature can cause rapid moisture loss, which significantly disturbs the development of the root system and eventually reduces fiber quantity and quality (Guo et al., 2024b). Moreover, the fatty acid composition of roots is susceptible to temperature deviation (Xu et al., 2022; Li et al., 2022a). Under heat stress, the membrane stability of cotton root cell, is often compromised leading to lessen cellular function and ions transportation due to membrane damage (Abro et al., 2024; Sheri et al., 2023; Lu et al., 2023). Many studies have reported the increased concentration of Reactive Oxygen Species (ROS) in cotton root cells under heat stress resulting in oxidative stress (Sekmen et al., 2014; Fan et al., 2022; Majeed et al., 2021). Heat stress also alters the cell wall composition and structure, reduces the mechanical strength and adaptability of roots to stress (Tsvetkova et al., 2002; Ezquer et al., 2020; Zhou et al., 2014).

Genetic variability is therefore of great importance in crop improvement, including cotton, as it provides valuable insights into the genetic architecture that can be used to enhance plant resistance to abiotic stresses. In cotton, genetic differences in the root systems are very important because they directly influence plant productivity. Superior genotypes with well branched root systems demonstrated a stronger ability to support plant growth. A robust and extensively branched root system can significantly enhance cotton yield, even under unfavorable moisture conditions. Till now few studies have identified the genes such as *GhNAC1* (Sivakumar et al., 2021; Kundu et al., 2019), *GhWRKY41* (Adjibolosoo et al., 2024), *GhHSP17.3* (Lv et al., 2024), *GhROD1* (Ding et al., 2024), *GhANN11* (Luo et al., 2024), *GhADF1* (Qin et al., 2022), *GhZFP1* (He et al., 2019), and *GhARF1* (Wang D. et al., 2024) associated with cotton root traits under heat stress. The up and downregulation of these genes under various heat stress conditions have confirmed the resistances in different cultivars (Abdullah et al., 2023; Ding et al., 2024). Meanwhile, the enrichment of the vascular system facilitates the growth of lateral roots, enhancing the plant’s ability to actively withstand abiotic stresses (Chen L. et al., 2023). Genotypes with dense root systems and well developed lateral root exhibit greater resilience under such conditions (Guo et al., 2024a). Therefore, identifying and utilizing genes associated with this vital trait can help develop high-yielding, stress tolerant genotypes (Nguyễn et al., 2024). Alternatively, tradition breeding approaches, such as crossing diverse genotypes, cab be employed to generate diverse germplasm with improved root system, enabling the crop to reduce severe climatic challenges.

### 2.2 Photosynthetic efficiency and stomatal conductance

Cotton growth also dependent on photosynthesis, a process highly sensitive to temperature fluctuations (Abro et al., 2024). The optimal temperature for photosynthesis is around 30°C; however, its efficiency declines rapidly with each degree rise in temperature (Sargent et al., 2024). Heat stress during the day adversely affects the rate of photosynthesis in cotton, leading to reduced yield and significant alterations in physiological traits, ultimately causing stunted growth and loss of boll formation (Ma et al., 2024; Snider et al., 2022).

Heat stress led to the deactivation of Rubisco, an essential enzyme for catalyzing carbon fixation during photosynthesis, by inhibiting Rubisco activase, a chaperone protein essential for maintaining Rubisco function under stress. This results in reduced photosynthetic activity in cotton (DeRidder and Salvucci, 2007). For example, Carmo-Silva et al. (2012) reported a reduction in stomatal conductance and net CO_2_ assimilation with increasing leaf temperature, which caused lower photosynthesis activity was observed due to Rubisco inactivation (Crafts-Brandner and Law, 2000). This deactivation of Rubisco is considered the prime limitation of photosynthesis at temperature around 40°C (Stainbrook et al., 2024). Heat shock proteins, which are responsible for or associated with developing tolerance against heat stress in plants, are closely association with a plant’s photosynthetic capability (Khan et al., 2022). Rehman et al. (2021) reported that the upregulation of *GhiHSF14* and downregulation of *GhiHSF21* in cotton under heat stress contributed to increased photosynthetic rates, thereby enhancing tolerance (Dilnur et al., 2019). Even mild heat stress significantly disturbs the photosynthetic activity of cotton by interfering with electron flow in the leaves. This adverse effect of high temperature on photosynthesis is irreversible due to the interruption in electron transport (Kamatchi et al., 2024).

Under high temperature, the fluidity of the thylakoid membranes increases, causing the light-harvesting complexes of photosystem II (PSII) to detach from the membranes (Haque et al., 2014). This disrupts the structural integrity of PSII and affect electron transfer (Haque et al., 2014). Heat stress also dissociates the oxygen-evolving complex of PSII, inhibiting electron transport to the acceptor side of PSII (Húdoková et al., 2022; Allakhverdiev et al., 2008). Recent studies have shown that heat stress induces oxidative stress in plants, generating ROS that damage the PSII reaction center and trigger a repair cycle (Pospíšil, 2016; Dev et al., 2024). Consequently, PSII is highly sensitive to heat stress (Pospíšil, 2016; Húdoková et al., 2022).

Heat stress brings alterations to essential metabolic processes in cotton, causing low productivity and shortened lifespans. In order to minimize water loss through evaporation, plants lower stomatal openings, which limits photosynthesis. Contrariwise, high stomatal permeability enhances evaporative cooling, alleviating heat stress by lowering leaf temperature (Wang et al., 2022a). Due to Rubisco (an important carbon fixation enzyme) inactivation during mild heat stress, stomatal permeability and net photosynthesis are reduced in many plant species (Marchin et al., 2022). Since stomata regulate both water evaporation and CO_2_ exchange, their role is crucial in developing varieties that could easily tolerate heat stress. Enhanced stomatal aperture in high-yielding genotypes might improve photosynthesis and transpiration rates under heat stress conditions (Marchin et al., 2022).

Furthermore, studies have found that more transpiration led to partial cellular membrane degradation in plants, a mechanism aimed at mitigating water loss (Li E. et al., 2022). Photosynthetic capacity and chlorophyll content as indicators in evaluating heat tolerance in cotton and wheat genotypes have been widely used, aiding in identifying the mechanism associated with genotypes adaptation to high-temperature environments. These findings highlight the importance of stomatal regulation and photosynthesis performance in developing heat-resilient crop varieties (Peng et al., 2022).

### 2.3 Reproduction

In general, higher night temperature in cotton leads to poor reproductive performance of the crop (Istipliler et al., 2024). Fertilization typically occurs 12–24 h after pollen release (Masoomi-Aladizgeh et al., 2021). Heat stress during the flowering stage can disrupt pollination, ultimately reducing the number of bolls (Majeed et al., 2021). Pollen grains are extremely susceptible to heat stress as compared to ovule, making them a major factor in reduced fertilization under heat stress (Khan et al., 2023a). Pollen grains require a significant amount of energy for their survival, but heat stress reduces carbohydrates production, adversely affecting their viability (Khan et al., 2022). This also affects the cotton photosynthetic capacity, increases respiration and photorespiration, and hinder the translocation of nutrients (Ahmed et al., 2023a; Naveed et al., 2023). Extremely higher temperature restricts the efficiency of fertilization by interfering with carbohydrate metabolism and calcium homeostasis (Khan et al., 2022). Moreover, increased oxidative stress in tissue can substantially reduce cotton yield (Zhou et al., 2014). Heat stress reduces both quantity and viability of pollen grains in cotton. The pollen tube development is particularly sensitive to heat stress, with its length decreasing substantially at 34°C and nearly ceasing at 43°C. A previous study showed that *GhCKI* genes are highly expressed in fully developed anthers and control infertility. Introducing the *GhCKI* gene into anthers of heat-sensitive cotton lines could mitigate infertility under heat stress (Min et al., 2013). This finding highlights the need of identifying and utilizing such stress-tolerant genes to enhance the resilience of cotton germplasm. Exploring and incorporating heat-resistance genes (Table 1) into breeding programs is essential to improve cotton’s adaptability and productivity in the face of climate change (Salman et al., 2019). Several genes such as *GhBEE1* (Chen et al., 2017), *ARFs* (Ding et al., 2017), *GSTU24* (Chen et al., 2018), and *MPS1* (Chen et al., 2018) regulate anther indehiscence. However, among the 88 reported genes, only five are involve in controlling carbohydrate metabolism and cell death (Singh et al., 2007). Exposure to heat stress has been linked to several pathways that lead to sterility. Enzymes associated with carbohydrate metabolism and transports play important role in heat stress. Such enzymes can serve as markers for assessing pollen viability under heat stress. Previous experiments have investigated the cause of sterility by overexpressing the *GhCKI* gene in cotton (Li et al., 2022c). Li M. et al. (2021) identified six nuclear genes (*WRKY28*, *ghi-MIR156*, *ghi-MIR171*, *AGL19*, *MAPKK6* and *ghi-MIR7484*) associated with anther abortion in cotton. The growth of the pollen tube and progeny development in plants mainly depends on the quantity of pollen grains deposited on the stigma (Stavert et al., 2020). However, the effects of restrictive pollination on progeny development are significant and require further exploration. A key aspect is the reduced pollen tube growth rate caused by a limited number of pollen grains (Li et al., 2023). When pollen grain availability is below optimal levels, phenotypic variation among progeny tends to increase. Studies have demonstrated a positive correlation between pollen grain quantity and the rate of pollen tube development (Reese and Williams, 2019). Kakani et al. (2005) studied the effects of different levels of heat stress, i.e., low, moderate, and high on the length and germination of pollen tubes in 12 cotton genotypes. The highest pollen tube length and maximum germination were observed at 32°C. However, at 44°C, no pollen tube formation was noticed and no pollen germination occurred at 47°C. Similarly, Burke et al. (2004) also reported that at 47°C the length of the pollen tube reduced significantly as temperature >32°C. The pollen germination percentage also decreased at a temperature of 37°C. Furthermore, an experiment was conducted to assess the effect of pollen quantity on pollen tube growth rate in cotton plants. In the first trial, almost 20 pollen grains were applied to the stigma of an emasculated cotton flower, while in the second trial, a large quantity of pollen grains was applied to the stigma of emasculated flowers (Ter-Avanesian, 1978). In the first trial, pollen tubes took 15 h to reach the ovules, whereas in the second trial, they required only 8 h indicating that the growth rate of pollen tubes in the second trial was nearly twice as fast (Ter-Avanesian, 1978). The slower growth of pollen tubes under limited pollen conditions could be attributed to the physiological association between the stigma and reduced number of pollen grains, potentially impacting nutrient availability and signaling pathways (Yang et al., 2022). Additionally, cultivars with longer anthers, which produce more pollen grains, have reported to exhibit greater resilience to heat stress, suggesting that pollen grain abundance may confer an adaptive advantage under adverse environmental conditions (Yang et al., 2022).

**TABLE 1:** Heat stress responsive genes reported in cotton.

Gene	Function	Reference
HSPCB	Heat shock protein calmodulin binding	Sotirios et al. (2006)
IAR3	Upregulated under both short- and long-term heat stress	Demirel et al. (2014)
AtCaM3	Key component in the Ca2+-CaM (calmodulin) Heat Shock signal transduction pathway	Liu et al. (2005)
ERD15	Augments stress tolerance by enhancing the efficiency of PSII	Ziaf et al. (2011)
DDF1	Regulating responses to heat stress	Kang et al. (2011)
GhiHSF14	Upregulated in heat stress	Dilnur et al. (2019)
GhHSF39	Immediate response to heat shock	Wang et al. (2014)
Rubisco activase genes GhRCAα1, GhRCAα2, GhRCAβ	Expression under heat stress	DeRidder and Salvucci (2007)
AtSAP5	Upregulates expression of heat stress responsive genes	Hozain et al. (2012)
AsHSP70	Heat shock protein from *Agave sisalana*	Batcho et al. (2021)
HSP70-17	Heat shock protein improving male fertility under heat stress	Khan et al. (2022)
GhMAP3K65	Increase susceptibility to heat stress	Zhai et al. (2017)
GhHS26 and GhHS97	Heat tolerant HSP genes	Ali et al. (2022)
GhHRP	Heat responsive protein	Abdullah et al. (2023)
GhSGT1 and GhSGT2	Showed different gene expression and enzymatic activity under heat stress	Li et al. (2014)
WRKY25	Heat responsive gene in *Arabidopsis thaliana*	Li et al. (2009)
GhWRKY3, 83 and 97	Expressed under heat stress	Dou et al. (2014)
AacCas12b	Temperature Inducibility	Wang et al. (2020)

### 2.4 Other developmental stages

Heat stress decreases leaf and leaf-related traits of cotton to a great extent. The reduction in leaf length, leaf width, leaf ratio, and leaf area depend on the time period and degree of heat exposure. These traits suffer more damage when exposed to prolonged and severe heat stress (Saleem et al., 2021; Sarwar et al., 2023; Yousaf et al., 2023). Leaf area plays a very important role in the process of photosynthesis as it captures most of the sunlight and is very sensitive to high temperature and works optimally at 26°C–28°C (Terashima et al., 2011; Wang Y. et al., 2023). Till now, many studies have identified genes such as *HSP70*, *HSP101*, *HsfA1*, and *HsfB1*, that are associated with leaf traits. These genes also contribute to enhancing heat stress tolerance in cotton (Ikeda et al., 2011; Zhou et al., 2023). Furthermore, ABA-responsive element binding factors have also been reported to regulate the expression genes responsible for heat resistance, thereby improving leaf morphology (Huang et al., 2016).

Flowering branches are also highly sensitive to high temperature with a significant decrease in the flowering ratio observed after prolonged exposure to temperature exceeding 42°C (Saud and Wang, 2022). When the daytime temperature rise above 30°C during the flowering period, it leads to the shedding of squares and flowers (Bange et al., 2022).

When temperature exceeds the limit of 28°C, it strikingly affects the seed number and boll size; however, few fruits remain at a temperature >32°C (Saini et al., 2023). Heat stress also influences the production of vegetative and flowering branches (Abro et al., 2023). When temperature increases from 30°C to 40°C, fruiting sites are reported to increase exponentially (Ahmad et al., 2020). However, the strength of bolls strikingly decreases above 35°C and approaches almost zero when the temperature rises above 40°C (Reddy K. R. et al., 1997). It is observed that newly developed cotton bolls are frequently shed when the average day temperature is > 32°C (Majeed et al., 2021).

### 2.5 Yield

The net yield of cotton is highly susceptible to heat stress, with numerous studies reporting a negative correlation between elevated temperature and cotton lint yield (Conaty et al., 2015). The annual variations in cotton yield are largely attributed to differences in temperatures during the growing season. Studies have shown that cotton lint yield decreases rapidly when temperatures exceed 32°C, and fruiting efficiency begins to drop at temperature above 29°C (Jans et al., 2021; Ashraf et al., 2023). Such heat stress suppresses photosynthesis, leading to low carbohydrates production, which is necessary for cotton fiber quality and final yield (Zahra et al., 2023).

Elevated temperature at night further exacerbates yield loss by enhancing respiration, which drop the level of carbohydrates (Abro et al., 2023). This reduction in carbohydrates adversely affects several key traits like seed setting, size of bolls, seeds per boll and quality and quantity of fiber (Soliz et al., 2008). Boll size and number, the primary contributors to cotton yield, are particularly vulnerable to heat stress. Boll retention, which directly associated with yield, decline significantly under heat stress (Patra et al., 2023). Studies have shown that heat stress declines the cotton plant capability of retaining boll, causing premature boll shedding and contributing to substantial yield losses globally (Saini et al., 2023).

Studies have also revealed that slight temperature changes may not affect seed weight, but they can significantly reduce the number of seeds per boll (Mudassir et al., 2021). To mitigate these impacts, breeding strategies are being developed to enhance heat tolerance of cotton. Reproductive tissues, which are more sensitive to heat stress, are recognized as key contributors to yield loss. Therefore, breeding programs focusing on improving the resilience of these tissues are critical for maintaining yield stability under heat stress (Majeed et al., 2021).

## 3 Heat-induced oxidative stress and cellular defense mechanisms

### 3.1 Antioxidant activity

Plants relay on tightly regulated oxidation-reduction reactions to balance energy generation and consumption, but these processes are profoundly influenced by environmental stressors, leading to significant alterations in their metabolic activities (Suzuki and Mittler, 2006). Such metabolic changes affect the concentrations of several biomolecules. Similar to humans, plants subjected to heat stress undergo metabolic dysregulation, resulting in an excessive accumulation of reactive oxygen species (ROS) inside of their cells (Suzuki et al., 2012). While ROS overproduction under heat stress can oxidative damage to cellular organelles, proteins, lipids, and DNA, it is important to note that ROS also play crucial physiological roles. At controlled levels, ROS are involved in key processes such as detoxification of hazardous compounds, antimicrobial phagocytosis, programmed cell death (apoptosis), and signaling pathways that regulates stress tolerance, cell growth, seed germination, root hair development, and cellular senescence (Considine et al., 2015; Singh et al., 2016).

ROS levels overwhelm the plant’s antioxidant defense systems under severe and prolonged heat stress, causing oxidative stress and irreversible damage to important cellular components (Mittler, 2002). ROS encompass a range of highly reactive molecules, including singlet oxygen (1O2), hydrogen peroxide (H_2_O_2_), and free radicals like the hydroxyl radical (OH) and superoxide anion (O2•-). These species are generated through cell-based mechanisms involving the excitation and reduction of molecular oxygen (O2), often triggered by disruptions in metabolic pathways and environmental challenges. For example, Lv et al. (2023) demonstrated the participation of H_2_O_2_ in regulating high intensity blue light (HB) induced hypocotyl phototropism in cotton under heat stress. Their findings revealed that exposing cotton seedling to HBL from one side results in uneven distribution of H_2_O_2_ and inhibits the elongation of hypocotyl cells. Understanding the dual role of ROS as both damaging agents under stress and essential signaling molecules highlights their complexity in plant stress biology.

The evaluation of cotton’s antioxidative scavenging ability and ROS concentration serves as a critical criterion for selecting heat-tolerant cultivars (Majeed et al., 2019). In a study involving two cotton cultivars, 30 days old seedlings were subjected to moderate heat stress, with temperature increased from 30°C to 45°C. During heat stress, lipid peroxidation increased by 40%–170% and hydrogen peroxide levels rose significantly by 206%–248%. The concentration of non-enzymatic antioxidants increased proportionally with the temperature. Enzymatic antioxidant activities, including superoxide dismutase (SOD), catalase (CAT), peroxidase (POX), and ascorbate peroxidase (APX), also increased by 56%–70%, 37%–69%, 43%–91%, and 22%–27%, respectively. These finding suggest that genotypic differences among cultivars influences ROS generation and antioxidants responses. Cultivars with higher antioxidant levels and lower ROS concentrations exhibited greater tolerance to heat stress (Loka and Oosterhuis, 2016).

In a separate study, cotton plants were grown under two temperature regimes; 38°C and 45°C. The results revealed no significant differences in H_2_O_2_ levels between the two temperatures. However, proline concentration decreased significantly and quickly as the temperature increased from 30°C to 45°C. While CAT, POX, and APX activities increased with rising temperatures, SOD activity dropped at a temperature of 45°C (Loka and Oosterhuis, 2016). Additionally, another study demonstrated that applying H_2_O_2_ topically to cotton plants activates SOD and CAT, supporting the idea that foliar H_2_O_2_ treatments can enhance heat tolerance in cotton without negatively affecting crop yield (Majeed et al., 2021).

Another study was performed to find out how high temperature at night affect the biochemical responses of leaves and pistils in an upland cotton cultivar. The results showed that as the nighttime temperature increased, glutathione reductase activity in the leaves rose significantly, whereas its concentration in the pistils and other floral components are less influenced by fluctuations in nighttime temperatures compared to leaves and other vegetative parts of the cotton plant (Sun, 2012). Yousaf et al. (2023) also revealed that the activities of biochemical attributes of upland cotton genotypes significantly increased under heat. SOD, POD and CAT increased by 52%–98%, 54%–169%, and 65%–74%, respectively. Heat stress also induced oxidative stress, as evidenced by a substantial increase in H_2_O_2_ levels from 7.1% to 28.7%. Correlation analysis also revealed that SOD and POD displayed positive, and CAT and H_2_O_2_ negative correlation with seed cotton yield. The results suggest that the antioxidant capacity of cotton genotypes plays a critical role in their heat tolerance, with BH-232 exhibiting the most effective biochemical response among the tested genotypes.

### 3.2 Small RNAs roles in regulating heat stress

Heat stress in cotton also cause various harmful effects at both the cellular and molecular levels including damage to DNA and proteins (Yadav et al., 2024). Heat stress disrupts cellular and molecular functions, particularly at the transcriptional and post-transcriptional levels. Transcriptional factors (TFs) play a vital role as key regulatory elements, influencing the expression and activity of multiple genes under heat stress (Yadav et al., 2024). MicroRNAs (miRNAs) have generated significant scientific interest due to their potential involvement in the precise regulation of TFs, which could impact stress responses and adaptation mechanisms (Wang et al., 2024c). Under heat stress conditions, miRNAs play an important role at the molecular level, bridging significant gaps in research between genetics and molecular breeding (Wang et al., 2024c). MiRNAs, also known as killer RNAs, are categorized as internal non-coding RNAs (ncRNAs) (Corona-Gomez et al., 2022). This category of RNA has profound effects on biological and metabolic processes. Derived from RNA hairpin precursors, miRNAs are approximately 21 nucleotides in length. These killer RNAs are processed by a specific double stranded RNA degrading enzyme, ribonuclease (Sharma et al., 2025).

The core components of the miRNA pathway under heat stress are highly conserved, with subtle variations distinguishing their roles in plants. miRNAs are primarily transcribed by RNA polymerase II (Pol II) as long primary transcripts known as pri-miRNAs which is then converted into a hairpin structure (Wang et al., 2024e). Pri-mRNAs are then further processed into a precursor miRNA (pre-miRNA) in the nucleus. Notably, approximately one-third of known miRNAs are embedded within the introns of protein-coding genes and are frequently co-transcribed with their respective host genes (Sharma et al., 2025). Additionally, some miRNAs originate from exonic regions or are derived from larger ncRNAs. The biogenesis of most miRNAs follows a sequential processing pathway involving members of the RNase III family, namely, Drosha and Dicer (especially Dicer like-1 enzyme) (Maurya et al., 2025). Following transcription, Drosha cleaves the primary transcripts in the nucleus, excising short hairpin structures (∼60–100 nucleotides) to generate precursor miRNAs (pre-miRNAs) (Asadi et al., 2024). These pre-miRNAs are then transported to the cytoplasm via Exportin 5 (XPO5), where Dicer further processes them into mature double-stranded RNAs (∼19–24 nucleotides) (Sumaira et al., 2024). Notably, some miRNAs deviate from this canonical biogenesis pathway and mature independently of Drosha processing. Such miRNAs include mirtrons and tailed mirtrons, which generate their precursor forms through splicing and exonuclease-mediated trimming. Subsequently, the mature miRNA associates with an Argonaute (AGO) protein to assemble the RNA-induced silencing complex (RISC) (Hameed et al., 2024). This complex then selectively binds to complementary sequences on target mRNA molecules, resulting in either mRNA degradation or translational repression, thereby modulating gene expression at the post-transcriptional level (Liang et al., 2023). The efficiency and precision of this regulatory pathway are orchestrated by key proteins, including HASTY, HYPONASTIC LEAVES1 (HYL1), and SERRATE (SE), which play essential roles in miRNA processing and export (Bielewicz et al., 2023). Furthermore, miRNAs also modulate HSPs expression under heat stress by directly binding to the 3′untranslated region (UTR) of HSP-encoding mRNAs. This interaction facilitates HSP upregulation either by enhancing translation or preventing mRNA degradation, thereby fine-tuning the cellular stress response. Through this regulatory mechanism, miRNAs ensure that HSP levels are appropriately adjusted in response to thermal stress, thereby safeguarding cellular homeostasis and preventing protein misfolding under adverse conditions (Islam et al., 2024).

The latest advances in high throughput sequencing have facilitated the precise identification of miRNAs across plant species including cotton (Naveed et al., 2025; Liu H. et al., 2014; Chen et al., 2020). Previous research has depicted that heat stress can induce the differential expression of specific miRNAs in different plant species (Dong et al., 2015; Kan et al., 2023). However, the mechanism remains largely unexplored in cotton. A comprehensive set of 77 miRNAs has been identified, including 33 previously known and 44 newly discovered miRNAs. Of these, 41 miRNAs exhibited differential expression under normal temperature, while 28 miRNAs displayed distinct expression patterns under heat stress (Zhang and Pan, 2009).

Computational analysis has been instrumental in characterizing various miRNAs families based on their conserved features across different developmental stages in cotton (Hamid et al., 2024). The functionality of these miRNAs has been validated through deep sequencing (Ahmed et al., 2024). In cotton tissues, several highly and lowly conserved miRNAs families have been identified, a process widely studied in *Oryza sativa* and *Arabidopsis* (Suddal et al., 2024; Mazhar et al., 2022). Despite this, there is a significant gap in the literature regarding this role of miRNAs in stress tolerance in cotton, which require further investigation. To address this, a comprehensive analysis of the cotton genome using high-throughput sequencing is underway worldwide. This approach is considered highly effective for detecting and classifying miRNAs and elucidating their roles under stress conditions, ultimately aiding in the development of superior cotton germplasm. The identification of miRNAs helps in detecting stress responsive genes (Fuck et al., 2024). Moreover, the linkage of miRNAs profiles with stress controlling networks offers valuable insights for manipulating plant genetic material to enhance tolerance to heat stress (Samynathan et al., 2023). This knowledge can be utilized to develop dominant or stress resistance cotton varieties capable of withstanding high temperatures and other abiotic challenges. Previous studies have shown that certain miRNAs are upregulated under heat stress (Zeng et al., 2023; Gan et al., 2023).

The Identification of heat-responsive miRNAs provide the basis for molecular breeding. Using advance techniques, miRNAs associated with various quality traits can be identified. Plant hormones, such as abscisic acid (ABA), assist the plants to survive under heat stress (Wang K. et al., 2024). ABA significantly influences plant developmental stages during stress by its regulatory functions and impact on signal transduction (Liu H. et al., 2022). Understanding ABA’s role and how it deviates under stress conditions will provide valuable insights for mitigating crop yield losses and clarifying its function in stress-induced signal transduction pathways (Cutler et al., 2010). ABA is synthesized from carotenoids through glyceraldehyde-3-phosphate and isopentenyl diphosphate in cells containing plastids, such as those in roots and leaves (Bergman et al., 2024). It is known as a growth-inhibitory hormone because it suppresses cell proliferation. Under water deficit conditions, ABA production increases significantly in roots and is subsequently transported to shoots. In leaves, ABA levels can rise by up to 50% under water stress (Taiz and Zeiger, 2002), where it induces stomatal closure to minimize water loss (Kaya et al., 2019). ABA has proven beneficial under drought stress by enhancing the hydraulic conductivity of plant root systems (Grover et al., 2013). During heat stress, plants rapidly accumulate endogenous ABA, which plays a vital role in improving heat resistance by regulating ROS levels (Li N. et al., 2021). Exogenous application of ABA has also been shown to mitigate the adverse effects of heat stress and enhance heat tolerance (Sumaira et al., 2024). Moreover, ABA significantly regulates heat stress transcription factors (HSFs) and heat shock proteins (HSPs), further strengthening plants' heat resistance. In earlier studies, transposon-mediated mutations were employed to investigate ABA-related responses in an Arabidopsis mutant known as *hyl1* (Ali and Yan, 2012).

Various studies have demonstrated that ABA treatment significantly influences the expression of miRNAs (Ali and Yan, 2012). Previous research has shown that the transcription factor ABI3 facilitates ABA in inducing and accumulating the expression of miRNA159 (Li et al., 2007). This induced miRNA159, in turn, upregulates the expression of other transcripts involved in ABA signaling. Several studies have explored the impact of ABA on transcription factors and miRNAs to highlight its importance under stress conditions across multiple crops (Hao and Zhang, 2022).

Elucidating the pivotal role of ABA and its molecular traits in greater detail can provide a clearer understanding of stress responses in cotton. Such insights would enable scientists and breeders to strategically manipulate existing germplasm for the benefit of humankind (Zeng et al., 2023). It is, therefore, a fundamental responsibility of scientists to enhance crop yields to meet the demands of the growing global population. The responses of miRNAs under heat and ABA stress were profiled using membrane arrays alongside controls. Replicated data from the membrane arrays under ABA stress were collected to obtain average responses (Zhang L. et al., 2023).

There are several challenges associated with the application of miRNAs in cotton and other crops to mitigate heat stress. Plant miRNA prediction has traditionally relied on pattern-based methods, which utilize small RNA sequencing data and biological criteria to identify authentic miRNAs (Yadav et al., 2024). However, these methods face several challenges and limitations when applying miRNAs to enhance plant resilience against heat stress. Key issues include the high false-positive rate due to sequence similarities, difficulty in distinguishing true miRNAs from degradation products, and the limited ability to predict novel miRNAs without prior reference sequences (Kuang et al., 2023). Additionally, these methods often struggle to capture the dynamic expression patterns of miRNAs under heat stress, making it challenging to identify stress-responsive miRNAs with functional significance in plant adaptation (Patra et al., 2023). Scientists introduced machine learning to overcome the limitations and challenges associated with pattern-based methods. Machine learning-based approaches have been widely employed for predicting plant miRNAs, leveraging algorithms trained on extensive datasets to analyze miRNA-target interactions (Fuck et al., 2024). These methods consider multiple factors, including sequence context, structural characteristics, and evolutionary conservation across species, often surpassing pattern-based approaches in accuracy (Sittaro et al., 2023). However, machine learning models still require refinement, as their predictions are not always entirely reliable. The advancement of deep learning techniques highlights the potential for developing innovative models that could replace existing tools and improve the accuracy of plant miRNA identification (Jafar et al., 2024).

Despite the progress made, training machine learning models is a complex process that demands meticulous design and implementation. Ensuring model reliability is particularly crucial when individuals without specialized expertise are involved (Tian et al., 2024). High-quality training datasets are essential for building effective models, making data collection a fundamental step (Liu et al., 2022b). A well-structured positive dataset consists of experimentally validated miRNAs sourced from public databases or published literature (Singla et al., 2024). The latest version of miRBase (v.22) includes 38,589 precursor miRNAs from 271 species spanning plants others and unicellular organisms (Kozomara et al., 2019). However, concerns have been raised about the reliability of many plant miRNA loci and families listed in miRBase, as some lack strong supporting evidence (Ražná et al., 2025). To address these data quality concerns, the Plant miRNA Encyclopedia (PmiREN) was developed as a more refined database, integrating updated annotation strategies to minimize biases found in earlier resources (Bambil et al., 2025). PmiREN enhances data accuracy by selectively incorporating genomic and small RNA sequencing data, ensuring higher-quality miRNA records. The latest PmiREN 2.0 release comprises 179 plant species 38,186 miRNA loci from 7,838 miRNA families (Guo et al., 2022). Despite these computational advancements, miRNA prediction remains challenging, with false positives being a persistent issue. Studies indicate that a significant proportion of plant miRNAs and miRNA families cataloged in miRBase may be inaccurately annotated, with more than a quarter of individual plant miRNAs and nearly three-quarters of miRNA families lacking sufficient experimental validation (Yan et al., 2024).

### 3.3 Heat shock proteins

HSPs are a diverse group of proteins that act as molecular chaperones, helping organisms survive under stress. They play a key role in protecting cells by stabilizing proteins, preventing damage, and assisting in refolding damaged proteins (Sable et al., 2018). The production of HSPs increases as temperatures rise. In cotton, HSPs are produced and accumulate at controlled temperatures between 38°C and 41°C (Farooq et al., 2023). These proteins are highly conserved across evolution and are present in both prokaryotes and eukaryotes.

HSPs are categorized into five main families based on their molecular weight: HSP20, HSP60/40, HSP70, HSP90, and HSP100 (Waters and Vierling, 2020). Each family plays a specific role in maintaining cellular balance and supporting different stages of plant development. Among these, small HSPs (sHSPs) are the most diverse, with low molecular weights ranging from 12 to 40 kDa (Silva et al., 2021). They vary widely in their location, function, and structure. sHSPs bind to unfolded proteins, preventing them from clumping together, and help in their refolding with the aid of ATP-dependent chaperones like ClpB/DnaK (Piróg et al., 2021). Most sHSPs contain an α-crystallin domain, which forms a double-ring structure (dodecamer) that assists in protein folding. Research has shown that the expression of sHSP genes, such as *Hsp17.7*, is closely linked to thermal stress tolerance in plants (Rehman et al., 2021). In cotton, quantitative analysis of the sHSP gene *GHSP26* revealed that its expression increases significantly during water deficit conditions, with a 100-fold rise in protein levels in the leaves (Fan et al., 2024).

HSP60, also known as chaperonin 60, is a mitochondrial protein that performs two critical functions during heat stress (Singh M. K. et al., 2024). First, it keeps proteins in an unfolded state for transport across the inner mitochondrial membrane. Second, it helps fold essential proteins within the mitochondrial matrix. HSP60 also supports photosynthesis-related proteins like Rubisco (Cömert et al., 2024). Studies have shown that mutations in the *Chaperonin-60α* gene, which codes for HSP60, lead to defective chloroplasts, resulting in poor seedling and embryo development in *Arabidopsis* (Kim et al., 2013). Deleting this gene causes cell death. Similarly, experiments with transgenic tobacco plants with reduced *Cpn60β* expression revealed issues like delayed flowering, stunted growth, and leaf yellowing (chlorosis) (Zabaleta et al., 1994).

HSP70 proteins play a key role in folding proteins and preventing their clumping. Increased HSP70 expression is a marker of heat tolerance in plants. In cotton, HSP70 genes are crucial for fiber development, and their inhibition leads to shorter fiber growth (Sable et al., 2018). This inhibition also causes oxidative stress by increasing H_2_O_2_ levels, damaging the ovule’s epidermal layer. HSP70 proteins also act as signaling molecules for activating or deactivating transcription processes (Ni et al., 2021).

HSP90 proteins differ from other chaperones because they are primarily involved in signal transduction, including working with signaling kinases and hormone receptors (Lubkowska et al., 2021). They also help fold proteins and are among the most abundant cellular proteins (making up 1%–2% of total cellular proteins) (Kumar et al., 2021). HSP90 proteins often work alongside HSP70 in a multi-chaperone system, and their expression rises significantly under heat stress HSP100 belongs to the AAA ATPase family and performs various functions, such as unfolding and breaking down protein aggregates (Lubkowska et al., 2021). Besides helping plants tolerate heat stress, HSP100 plays a role in basic cellular processes, including chloroplast development (Zeng et al., 2021).

All living organisms possess the ability to respond to environmental stresses, with molecular-level changes leading to a rapid surge in protein synthesis due to alterations in gene expression. These proteins, known as HSPs, stress proteins (SPs) or stress-induced proteins (SIPs), play a crucial role in stress adaptation (Banerjee et al., 2025). Heat stress disrupts essential cellular metabolic processes, including DNA replication, RNA transcription, mRNA export, and protein translation, causing a temporary halt until the cell stabilizes (Zheng et al., 2025). High temperatures significantly impact plant structure and metabolism, particularly affecting cell membranes and critical physiological activities. The enzymes involved in these processes exhibit temperature dependency, as their activity is influenced by the Michaelis–Menten constant (Raimanová et al., 2024). To survive heat stress, plants deploy various adaptive mechanisms, such as maintaining cell membrane integrity, scavenging ROS, synthesizing antioxidants, accumulating osmoprotectants for osmoregulation, and activating kinases, including calcium-dependent protein kinases (Kumar et al., 2024a). These processes facilitate increased transcription and signal transduction for chaperone proteins. Heat stress responses (HSRs) regulate signaling pathways by activating ABA-responsive genes, Ca^2+^-dependent signaling cascades, the synthesis of rapidly inducible osmolytes, ROS detoxification, and HSP-mediated protein folding (Wang K. et al., 2024). Upon detecting heat stress signals, plants initiate complex intracellular signaling cascades, which help regulate the activation of heat-shock transcription factors (HSFs) and HSPs, along with other stress-induced genes, to mitigate heat-induced damage (Yurina, 2023).

Genes encoding various HSPs are localized in different cellular compartments, including the cytosol, endoplasmic reticulum, chloroplast, mitochondria and nucleus (Kumar et al., 2024b). The accumulation of HSPs in these organelles is influenced by the severity of heat stress. For instance, nuclear HSPs localize in the cytosol at temperatures of 27°C and 43°C, while chloroplastic HSPs accumulate at around 37°C (Daniel et al., 2008). The transcriptional regulation of HSPs in response to heat stress is termed the heat shock response (HSR). This process is governed by HSFs, which bind to cis-regulatory elements called heat shock elements (HSEs) in the promoter regions of HSP genes (Hao and He, 2024). HSFs are categorized into three types, HSFA, HSFB, and HSFC: each with distinct roles. Among them, HSFA is a key regulator of the HSP cycle and exists in a monomeric form within the cytosol under normal conditions. Its activity is negatively regulated by HSP90, which maintains it in an inactive phospho-protein state (Rabuma and Sanan-Mishra, 2025). Upon heat stress, this repression is lifted as HSP90 dissociates, leading to the formation of a functional HSFA trimer. This activated trimer binds to HSEs in the promoter region, initiating transcription and subsequent HSP synthesis (Chen et al., 2024). HSFA1 serves as the primary regulator of this process, while HSFA2 shares structural and functional similarities with HSFA1 but is expressed only under stress conditions (Peng et al., 2025; Pan et al., 2024). Under extreme stress, HSFA2 forms a highly efficient hetero-oligomer complex with HSFA1, enhancing its regulatory function. This complex not only controls downstream heat stress-related HSP genes but also activates protective enzymes such as APX, POX, GR and GST reinforcing the plant’s defense mechanisms (Pandey et al., 2024).

### 3.4 Epigenetic regulation under heat stress

DNA methylation (DM) is a highly intricate mechanism that requires the involvement of various enzymes and cofactors. It begins when a DNA methyltransferase enzyme identifies a CpG dinucleotide (Ravi et al., 2025). This modification influences chromatin structure, ultimately leading to gene transcription suppression, as DM plays a crucial role in regulating gene expression during plant growth and responses to stress (Zhao et al., 2024). It is responsible for controlling key plant characteristics, including leaf morphology, resistance to diseases, and adaptation to environmental stressors. DM is vital for maintaining genome stability and modulating gene activity in plants (Talarico et al., 2024). The addition of a methyl group to the cytosine base results in the formation of 5-methylcytosine (5 mC), which participates in essential biological functions such as genome integrity, transcriptional silencing, developmental processes, and responses to heat stress (Wojciechowski et al., 2024; Neto et al., 2024). Acting as a repressive marker, 5 mC inhibits gene expression, with its levels controlled through both methylation and demethylation mechanisms. DM can occur through either active or passive pathways, and modifying its patterns has the potential to improve crop productivity and resilience to heat stress (Lawson et al., 2025).

Histone modifications primarily occur at the amino acid residues on histone tails and are mediated through processes such as methylation, phosphorylation, acetylation, and ubiquitination (Sharma et al., 2023). These modifications play a critical role in regulating gene transcription by modulating chromatin structure, either promoting an open chromatin state conducive to transcription or inducing a closed, repressive state (He et al., 2022; Correia et al., 2013). Specifically, histone marks like *H3K4me, H4K5ac* and *H3K36me* are associated with chromatin opening and active transcriptional regulation (He et al., 2022). Numerous studies have emphasized the pivotal role of histone modifications in epigenetic regulation and their contribution to stress responses, including the development of heat stress resistance in plants (Perrella et al., 2022; Nishio et al., 2024; Yáñez-Cuna et al., 2014).

Diverse plant species that undergo histone modification become susceptible to heat stress (Zhao et al., 2020). Previous studies have shown that heat stress effect transmission vector like *Chlamydomonas*, resulting in a significant increase in H3/H4 acetylated histones (Moler et al., 2019; Rommelfanger et al., 2021). Additionally, it has been demonstrated that heat shock can trigger the acetylation of histones (H3/H4) through specific transcriptional factor such as HSF1 (Weng et al., 2006). However, in forest plants such as oak cork, heat stress causes the decrease in amount of acetylated histone H3 (Weng et al., 2006). The increase in deacetylated histone H3 leads to suppression of chromatin in promoter region and cause failure of gene functionality (Xia et al., 2020). Therefore, to withstand heat stress, histone modification in the cell is essential for the production of cotton anthers.

The exact mechanism by which histone modifications influence gene expression in response to plant stress are still being explored. However, research on various plant species has led to several proposed mechanisms (Miryeganeh and Armitage, 2025; Abdulraheem et al., 2024; Acharjee et al., 2023; Millán-Zambrano et al., 2022). One such mechanism suggests that specific transcription factors or co-regulators are recruited to stress-responsive genes, guided by the recognition of distinct histone modifications in response to different environmental conditions (Dhatterwal et al., 2024).

The performance of TFs is accelerated in the presence of enhancers. The binding of enhancers to specific regions causes histone alternations, leading to increased transcription of relevant genes (Liu et al., 2023). Multiple studies have been carried out to understand the role of enhancers in gene functionality and developing resistance to heat stress, but much remains unknown (Giresi and Lieb, 2011). Enhancers are genomic segments involved in gene function studies and contains sequence motifs. These short sequence motifs serve as binding sites for the attachment of TFs (Zhao H. et al., 2022). Though, the relation between the motif sequence and activity of enhancers is unknown till now.

The relationship between sequence motifs and enhancer performance, as well as gene expression, needs to be better understood. Further research is required to explore the association between TFs and enhancer, and their role in opening of chromatin material (Hasanuzzaman et al., 2013). A positive and strong association between TFs and enhancer is crucial for higher gene expression (Andersson and Sandelin, 2020). This association is directly linked to histone modifications, ultimately leading to increased transcription of desired genes. Hence, it is essential to understand the linkage between enhancer and gene transcription in cotton to withstand harsh environmental conditions like heat stress (Wang R. et al., 2024).

Enhancers are often underestimated for their role in gene transcription. Enhancers comprised the sequences complementary to the TFs, but the relationship between the TFs sequences and enhancers in chromatin opening o remains unclear and needs to be elaborated (Panigrahi and O’Malley, 2021). Multiple methodologies have been developed, such as formaldehyde assisted selection of regulatory elements, to investigate the relation between active enhancers and DNA sequences/regions (Seuter et al., 2020). Techniques like FAIRE-seq, along with high-throughput sequencing and DNase-seq, are used to identify the positions of enhancers and promoters on chromosomes (Peng et al., 2017). Past studies have shown that enhancing dinucleotide repeat motifs (DRMs) can boost the activity of enhancers, which are wide distributed (Peng et al., 2017).

TFs play a crucial role in chromatin structure, which is essential for understanding gene functionality. In silent chromatin, specific group of TFs, such as pioneer TFs, play important role in determining the fate of the cell (Raccaud and Suter, 2018). This specific group of pioneer TFs was thought to be involved in reprogramming of the cell and have the capability to identify and engage genes that were developmentally silenced. In inactive (closed) chromatin, silenced genes are suspected to be present (Lai et al., 2018).

Applying epigenetics modifications to enhance cotton and other crop species resistance to heat stress presents several challenges and limitations. Due to variations in the composition of the epigenome among species, research has predominantly focused on specific epigenetic processes within different plant species (Langfordet al., 2024). In certain plants, the primary emphasis has been on DM, followed by histone modifications. Conversely, in species where DM levels are low or absent, studies have centered on non-coding RNAs or histone modifications alone (Liu and Zhong, 2024; Ding et al., 2022; Budak et al., 2020). However, these epigenetic mechanisms are interlinked. For example, in plants, the presence of *H3K4me3* and *H3K4me2* (Sharifi-Zarchi et al., 2017; Beacon et al., 2021) is typically associated with unmethylated DNA, whereas *H3K36me3* (Lam et al., 2022) is linked to the presence of DNA methylation. Additionally, in germ cells, histone modifications are believed to play a role in guiding DNA methylation machinery (He et al., 2011). Similarly, ncRNAs contribute to epigenetic regulation by recruiting binding proteins, influencing histone modifications, and ultimately affecting DNA methylation (Dávalos et al., 2019). A major limitation in current epigenetic research is the lack of focus on the interactions between these interconnected mechanisms.

The complexity and stability of epigenetic modifications in another major limitation. Epigenetic modifications, such as DM and histone alterations, are dynamic and can be reversible, making it challenging to maintain these changes over time and across generations (Manav et al., 2024). While some epigenetic changes can be inherited, the stability and consistency of these modifications across generations remain uncertain, affecting their potential use in breeding programs (Cao and Chen, 2024). Moreover, the selection of proper model plant to investigate the research question properly in epigenetics is another challenge (Kakoulidou et al., 2021). For example, *Arabidopsis thaliana* is one of the plant models of choice to investigate epigenetic regulations and modifications (Zhang et al., 2023). Selecting crops with less generations and complex epigenetic regulations makes it difficult to predict the outcomes and gain a clear understanding of the results. To gain a more comprehensive understanding of epigenetic regulations and mechanisms in nature, research should slightly extend beyond model species to a broader range of organisms. Of course, the selection of species must be carefully aligned with the research objectives, as it directly influences the availability of epigenetic mechanisms and the number of generations required to observe transgenerational effects (Gallusci et al., 2023). To avoid conducting blind experiments or working with non-model plant species that may be costly and challenging to maintain, an initial approach could involve comparative studies using existing data. This would help identify both common and unique epigenetic mechanisms, which could then be further explored in other species of interest (Doddavarapu et al., 2024).

## 4 Role of breeding, molecular breeding and omics approaches in developing heat tolerant cotton

Cotton cultivars often endure extreme heat, reaching temperatures as high as 50°C during May and June more than 20°C above the optimal range for healthy growth. This extreme heat significantly impacts crop yield, making the development of heat-tolerant cotton varieties a pressing need (Saud and Wang, 2022). Identifying traits that enable seedlings to resist heat stress is challenging due to their complex and dynamic responses (Abro et al., 2024). Researchers are actively exploring strategies to manage plants in high temperature conditions, including pharmacological, metabolic, and genomic adaptations (Li et al., 2022c). For example, Abro et al. (2015) developed fifty-eight cotton lines and screened them for heat stress in the field. Heat-tolerant genotypes were identified based on morphological traits, physiological traits, cell injury levels, and susceptibility indices. Seventeen out of the 58 genotypes were found to be highly heat-resistant. Developing enhanced heat resistance genotypes could improve yield and fiber quality in regions prone to high temperatures. Field-based methods are widely employed to assess heat tolerance, offering valuable insights into plant behavior in natural ecosystems. However, such studies face limitations in controlling environmental variables compared to controlled experimental setups (Khalid et al., 2023).

### 4.1 Conventional and molecular breeding

Traditional breeding for heat-resistant crops primarily relies on selection and genetically diverse germplasm under high-temperature testing environments to identify lines with superior yields (Nadeem et al., 2024; Majeed et al., 2021). However, improving cotton lines through conventional breeding under heat stress conditions often reduces yield losses. In regions with high temperatures, genetic lines are carefully selected at critical growth stages to ensure resilience. Germplasm evaluation is a fundamental step in breeding for stress tolerance, and numerous studies have focused on identifying heat-tolerant genotypes from existing genetic resources (Sharif et al., 2024; Ahmed et al., 2023b; Ali Z. et al., 2023).

Additionally, the use of crop wild relatives in plant breeding is gaining traction due to their unique traits, often absent in domesticated cultivars. These traits are commonly associated with resistance to biotic and abiotic stresses. Screening wild species and related relatives is highly recommended to expand the genetic diversity of breeding programs (Bohra et al., 2022). While transferring genes from wild species to cultivated crops can be challenging and often requires recombinant DNA technology, advancements in plant sciences have made it increasingly feasible to transfer genes across species boundaries (Kashyap et al., 2022). Once a desirable gene or trait is identified, it must be transferred into a suitable genotype or the selected plant purified through breeding. Classical methods such as single-plant selection, bulk selection, and pedigree selection remain widely used in cotton breeding (Munaweera et al., 2022). These traditional approaches, combined with molecular breeding tools, enable faster and more efficient screening and genetic improvement of cotton lines.

Transfer of a desirable gene(s) from one plant to another to improve a particular trait has also been widely used in almost all plant species, including cotton. Several traits in cotton including resistance to heat stress have been improved through different transgenic approaches. Recently, Batcho et al. (2021) applied Agrobacterium mediated technique to transferred *Agave sisalana* extracted *AsHSP70* gene in cotton to develop resistance against heat stress. The transformed gene exhibited highest expression in all tissues and improve physio-biochemical attributes significantly under heat stress. Similarly, Zhang L. et al. (2021) identified a gene called *SikCuZnSOD3* (associated with resistance to salt, drought, and elevated temperature) and transferred it to cotton using an agrobacterium-mediated technique. The transgenic cotton lines showed better growth with enhanced sugar, proline, water, and antioxidant content than regular cotton under stress conditions. Moreover, Esmaeili et al. (2021) revealed that the transfer of both *OsSIZ1* and *AVP1* genes in cotton improved cotton lint yield with an improvement in photosynthesis rate. Before heat stress, the photosynthesis rate in transgenic lines was about 72% as compared to non-transgenic lines, and this percentage increased to 108% under heat stress. Another HSP called *AtHSP101* (discovered in *Arabidopsis*), which is responsible for resistance to heat stress, enhanced pollen tube length and germination percentage in cotton upon transformation under heat stress as compared to non-transgenic lines (Burke and Chen, 2015). Developing heat resistance in cotton at the vegetative and reproductive stages is therefore essential to enhance yield. Furthermore, the transfer of the *Arabidopsis AtSAP5* gene into cotton improved overall plant growth and carob intake under combined heat and drought stress. These genetic improvements offer promising solutions for sustainable cotton production in extreme climates.

Besides conventional breeding, several other molecular breeding approaches such as marker-assisted breeding (MAB) and biotechnological tools have been discovered that are speedy and more accurate (Hassan et al., 2023). So far, several markers such as AFLP, RFLP and RAPD have been used across different crop species to identify the genotypic markers associated with abiotic stresses (Kundu et al., 2024; Gocer and Kulak, 2023). However, SNPs and SSR are currently the most widely used markers to identify QTLs associated with heat stress and other abiotic stresses in plants. For example, Rani et al. (2022), identified 26 linkage groups along with 175 marker loci in cotton heat resistant and susceptible genotypes. They identified about 17 QTLs that were strongly associated with 23 different morpho-physio heat resistant traits of cotton. The studied QTLs associated traits not only identified the heat resistant genotypes but also determined the most important traits to be focused on during developing heat resistant cotton lines. Demirel et al. (2014) also conducted an experiment to identify the marker QTLs associated with heat resistance in cotton by sequencing 25 expressed sequence tags (ESTs). During heat stress the expression of two markers, namely, *IAR*_*3*_ and *FPGS*_*3*_ were upregulated confirming the resistance to heat stress. Furthermore, *GhHS126* and *GhHS128* (non-annotated ESTs) were also found to be upregulated during heat stress. Another study was conducted to investigate the genetic based heat tolerance mechanisms of cotton. The expression level of few genes was quantified using real time PCR. Genes belonging to four different groups, i.e., transcription factors (*HSFA2* and *HSFA1b*), HSP (*GHSP26*, *HSP70-1*, and *HSP101*), calcium signaling (*ANNAT8*) and antioxidant activity (*APX1*). The expression level of all genes increased significantly under heat stress across different plant traits at seedling levels such as leaves, roots and ovaries confirming resistance to heat stress (Zhang Jin et al., 2016). Shaheen et al. (2009) identified 21 heat resistance SNPs marker located on different chromosomes across different cotton species including *Gossypium herbaceum*, *Gossypium laxum*, *Gossypium arboreum*, *Gossypium aridum*, *Gossypium stocksii*, *Gossypium gossypioides*, and *Gossypium sturtianum*. These SNPs can be useful in developing heat tolerant cotton and other crop varieties in a short period of time.

### 4.2 Genome editing (GE)

Although latest biotechnological/transgenic techniques are efficient, however due to their complicated protocols and lengthy procedures, prolong incubation period, the rate of transformation is comparatively low in case of cotton (Wang et al., 2022b). The CRISPR/Cas9 system has two subunits/parts 1: a clustered regularly interspaced palindromic repeat and 2: associated protein known as Cas9, the source of both the components is a bacteria named as *Streptococcus* pyogenes (Synefiaridou and Veening, 2021; Ali A. et al., 2023; Basu et al., 2023). This system is predominantly used for gene editing in plants to induce resistant against different stresses. It has been efficiently used for genome editing of many model plants (Kumari et al., 2024; Qurashi et al., 2024).

The use of CRISPR/Cas9 was found successful in case of standard/model plant, i.e., *Arabidopsis* (Belhaj et al., 2015). However, previous reports have confirmed the successful targeted genome editing of the cotton genome. Multiple genome editing has also been performed in allotetraploid cotton through targeting of *GhARG* and *GhCLA1* genes simultaneously (Shan et al., 2013). The main regulatory elements of genes are cis-sequences which is responsible for stress response (Liu J.-H. et al., 2014). These sequences also play important role in regulation of stress. To increase the tolerance of abiotic stresses and for the development of desirable QTLs to achieve phenotypic/genotypic variations of interest cis-sequences are targeted in CRISPR/Cas9 system (Zafar et al., 2020). However, there are certain technical hurdles and low efficiency of desirable transformation. Its use is limited to the cotton crop and needs to be further developed (Shan et al., 2013).

A comprehensive examination of the expression patterns of heat stress responsive genes in cotton subjected to prolonged periods of high temperatures demonstrated a notable increase in the expressions of *IAR*_*3*_, *TH1*, *FPGS*, *HS128*, and *HS126* genes. Conversely, the expressions of *RPS14*, *LSm8*, *CTL2*, *CIPK*, and *ABCC3* genes were observed to be downregulated in response to the heat stress conditions (Demirel et al., 2014). Hence, the precise manipulation of these genes that are overexpressed or under expressed in cotton using the CRISPR-Cas system presents an intriguing prospect for addressing the detrimental effects of heat stress. Additionally, several TFs and HSPs linked to heat stress-responsive genes have been suggested as promising candidates for enhancing plant heat tolerance (Ahmed et al., 2024). Utilizing the advanced CRISPR/Cas9 system, scientists have effectively manipulated the heat-stress sensitive albino-1 (*HSA1*) gene in rice, resulting in increased heat vulnerability when compared to natural plant variants (Qiu et al., 2018). Additionally, in the investigation of facultative parthenocarpy, the slagamous-like 6 (*Slagl6*) gene was identified as a potential candidate. Through precise alterations made to the *SlAGL6* gene using CRISPR-Cas9 technology, researchers have successfully produced heat-tolerant parthenocarpic tomato fruits (Klap et al., 2017).

The CRISPR activation system can selectively activate positive gene regulators associated with TFs and stress-related HSPs with remarkable specificity. On the other hand, negative regulators can be effectively suppressed using the CRISPR interference system (Kumari et al., 2024). In a particular study, researchers utilized the CRISPR activator and interference systems to modulate the expression of the *BZR1* gene. The findings revealed that upregulation of the *BZR1* gene through the CRISPR activator system led to an increase in H_2_O_2_ production and improved heat tolerance in rice. Conversely, plants with suppressed expression of the *BZR1* gene exhibited impaired H_2_O_2_ production in the apoplast and reduced heat tolerance. These results highlight the potential of the CRISPR activation and interference systems in regulating gene expression to influence stress responses and enhance desirable traits in plants (Yin et al., 2020). Until recently, the functions of *MAP3Ks* in cotton were not well comprehended. However, recent findings have shed light on their significance. It has been revealed that the expression of the *MAP3K65* gene is stimulated by various signaling molecules, pathogen invasion, and heat stress. This particular gene exacerbates susceptibility to pathogen infections and heat stress by exerting negative control over growth and development-related processes. Intriguingly, when *GhMAP3K65* was silenced, it resulted in an increased resistance to both pathogen infections and heat stress in cotton. Consequently, *GhMAP3K65* emerges as a promising target gene for the application of the CRISPR-Cas9 genome editing system, which could facilitate the engineering of heat tolerance in cotton (Zhai et al., 2017). *GhCKI* has been identified as a negative regulator of male fertility in upland cotton under heat stress. However, traditional genetic modifications of *GhCKI* result in male sterility, limiting its potential use in breeding programs. Li et al. (2025) introduced controlled variations in anther heat tolerance traits by developing weak promoter alleles of *GhCKI* using CRISPR/Cpf1 and CRISPR/Cas9 genome editing techniques. As a result, they characterized and identified two novel upland cotton lines with enhanced heat resistance, attributed to modifications in the *GhCKI* promoter. Further analysis demonstrated that the improved heat stress tolerance in these mutants is due to the inability of trans-acting factors including GhMYB4 and GhMYB73, which normally upregulate *GhCKI* under heat stress, to bind and activate its expression. This study presents an efficient approach for generating advantageous alleles while also providing valuable germplasm resources and molecular insights for breeding heat-tolerant crops. In another study, Khan et al. (2023b) investigated the impact of heat stress on male sterility in cotton, revealing that heat stress suppressed the expression of *GhAOC2*, a key gene in the jasmonic acid (JA) biosynthesis pathway. This suppression led to male sterility and reduced JA levels in the heat stress sensitive cotton line H05. However, applying methyl jasmonate (MeJA) to early buds restored anther fertility. To further understand the role of *GhAOC2* in JA biosynthesis and its involvement in the anther response to heat stress, CRISPR/Cas9 gene editing was used to create *Ghaoc2* knockout cotton plants. The mutant lines exhibited male sterility with reduced JA levels in anthers at anther dehiscence stage (ADS), tapetum degradation stage (TDS) and tetrad stage (TS). MeJA application to early-stage mutant buds (with TDS or TS anthers) restored pollen viability and anther dehiscence, while ROS accumulation in ADS anthers was reduced. These results suggest that heat stress induced suppression of *GhAOC2* disrupts JA biosynthesis, leading to excessive ROS buildup and male sterility. Their study highlights the exogenous JA application as a potential approach to improving male fertility in cotton under heat stress. Moreover, plants continuously perceive and adapt to changing light and temperature conditions throughout the circadian cycle. However, the molecular mechanisms governing plant adaptability under warm daytime conditions remain largely unknown. Abdullah et al. (2023) uncovers the role of *GhHRP* (protein associated with response to heat stress) in regulating cotton survival and adaptation under heat stress by influencing phytohormone signaling. According to their results, increased ambient temperatures enhance the binding of the phytochrome interacting ethylene-insensitive 3 (*GhEIN3*) and factor 4 (*GhPIF4*) complex to the *GhHRP* promoter, leading to elevated *GhHRP* mRNA levels. Overexpression of *GhHRP* promotes temperature-dependent accumulation of *GhPIF4* transcripts, facilitating hypocotyl elongation by activating heat responsive growth-related genes. Notably, upregulation of the *GhPIF4*/*GhHRP* complex enhances plant growth by regulating the expression of *A. thaliana* auxin biosynthetic gene *AtYUC8* and *AtACS8*, thereby fine-tuning the auxin-ethylene balance and reducing ethylene biosynthesis. *GhHRP* further protects chloroplasts from photo-oxidative stress by suppressing *AtACS7* and *AtACS8* while enhancing *AtYUC8*, HSP20 and HSP70 and heat shock transcription factor (*HSFA2*). Interestingly, the mutant exhibited impaired production of YUC8 and HSP, resulting in a contrasting phenotype with reduced ability to respond to high temperatures. These findings highlight *GhHRP* as a crucial heat-responsive signaling component that enables plants to adapt to temperature fluctuations by modulating auxin signaling, ensuring continued growth during warm conditions. Recently, Yu et al. (2023) developed an optimized CRISPR–dCas9–6×TAL-2×VP64 (TV) system to achieve controlled and strong activation of target genes in cotton. Various transcriptional activators, including EDLL, TAL, VP64 and were fused with dCas9 in different configurations, and their effectiveness in activating the LUC (*pGhTULP34-luciferase*) reporter gene was evaluated in tobacco. Multiple sgRNAs were designed, with sgRNA19 being selected for screening transcriptional activation domains. The efficiency of LUC transcription activation varied significantly among different combinations, with the dCas9–TV fusion demonstrating the highest activation efficiency, reaching up to a 51.9-fold increase.

Zinc-finger nucleases (ZFNs) and transcription activator-like effector nucleases (TALENs) have been utilized to carry out a diverse range of genetic modifications (Sprink et al., 2015). These techniques achieve this by introducing DNA double-strand breaks, thereby triggering two repair mechanisms: error-prone non-homologous end joining (NHEJ) or homology-directed repair (HDR) at precise genomic sites. This broadens the scope of genetic alterations that can be accomplished (Gaj et al., 2013). ZFNs are a type of targeting reagents comprising two essential components: zinc-finger-based DNA-recognition modules and the DNA cleavage domain derived from the FokI restriction enzyme (Carroll, 2011; Zhang et al., 2018). The recognition and binding process of zinc fingers involves each individual zinc finger identifying and attaching to a nucleotide triplet. These zinc fingers then come together to form groups that bind to specific DNA sequences. However, designing ZFNs with a strong affinity for a particular sequence is challenging, and they often exhibit a high rate of off-target effects (Cui et al., 2021). The journey from the development of ZFNs to the groundbreaking achievement of the first ZFN-based plant genome editing spanned a period of 9 years (Lloyd et al., 2005).

### 4.3 Genome wide association studies (GWAS)

Recent advances in sequencing technologies have allowed the plant breeders and geneticist to explore the underlying genetics of complex plant traits. GWAS detects millions of genetics variants called SNPs across plant genome to identify association between genotype and phenotype (Ahmed et al., 2024). GWAS offers a convenient alternative to the challenges posed by screening extensive biparental mapping populations. Consequently, GWAS has gained widespread application across various research studies (Nie et al., 2016), to identify quantitative trait nucleotides (QTNs) for quantitative traits (Spindel et al., 2015). Over the past decade, extensive research has focused on exploring the genetic correlations between SNP markers and phenotypes in order to identify candidate regions within the genome. This approach has allowed for the identification of potential QTLs and causal genes through high-resolution mapping facilitated by linkage disequilibrium (LD) analysis (Liu et al., 2018). These advancements have greatly contributed to our understanding of the genetic foundations underlying numerous traits in crop species (Phan et al., 2019; Tian et al., 2020).

GWAS has garnered substantial recognition and success in the field of human genetics, especially when combined with advancements in sequencing technologies. In recent times, GWAS has emerged as an invaluable tool in the study of crop plants, facilitating the identification of natural genetic variations that underlie the intricate characteristics of these agricultural species (Gupta et al., 2014). Association mapping is typically conducted using SNPs markers in combination with the phenotype of interest. By employing DNA sequencing, SNPs can be detected within diverse individuals or plants, and a comparison of their DNA sequences uncovers shared genetic variations in the genome (Su et al., 2024).

A thorough GWAS has successfully detected 4,820 genes associated with 13 fiber-related traits in cotton, offering valuable genetic reservoirs for enhancing fiber quality and performance (Ma et al., 2018). Through the examination of transcriptome variations and the utilization of GWAS, three specific loci associated with thermal tolerance were successfully detected. These loci encompass a total of 75 protein coding genes and 27 long noncoding RNAs. Moreover, expression quantitative trait loci (eQTLs) were identified for a remarkable 13,132 transcripts, shedding light on the relationship between gene expression and thermal tolerance (Ma et al., 2021). Recently, Han et al. (2022a) identified a total of 30,089 eQTLs laid on around 10 thousand genes while performing GWAS. Only 19 candidate genes such as *Ga14G1716*, *Ga14G0186*, *Ga13G2529*, *Ga13G1949*, *Ga13G1920*, *Ga13G1306*, *Ga08G1884*, *Ga01G1789*, *Ga04G1991*, *Ga11G2943*, *Ga10G2229*, *Ga10G0833*, *Ga08G2627*, *Ga08G1871*, *Ga02G0149*, and *Ga08G1873* distributed across chromosome 1, chromosome 5, chromosome six and chromosome eight were identified to be strongly associated with salt and heat resistance in cotton. Several other studies have also reported different SNPs, genes and alleles that are associated with different cotton traits and can be used to improve cotton resistance to various abiotic stress.

There are several challenges that limit the application and power of GWAS in detecting true associations between genetic makeup and observable traits. Variation in phenotypic data: before conducting GWAS, it is essential to carefully analyze phenotypic data and identify any outliers (Xiao et al., 2022). Significant variation in the data can reduce GWAS accuracy and may lead to false-negative or false-positive associations (Jiang et al., 2025). If outliers are present, their impact on GWAS should be assessed before proceeding. A boxplot is often used to visualize data and check for outliers, which should be removed if they are extreme (Rolling et al., 2025). However, removing outliers must not significantly alter overall phenotypic variance, as this is crucial for establishing associations. Once the data is filtered, traits with moderate to high heritability should be prioritized, as heritability indicates the extent to which genetic factors influence the phenotype (Gesteiro et al., 2025). The size of the study population is a second critical factor in GWAS, as reliable results depend heavily on sample size. A larger population improves the chances of detecting true associations, overcoming rare variants, and maintaining an appropriate frequency within the population (Tibbs Cortes et al., 2021). Typically, a sample size between 100 and 500 (or more) individuals is suitable for GWAS, while smaller samples (fewer than 100) may weaken the study’s power. Selection of individuals for GWAS often depends on specific traits of interest, genetic background, growth patterns, biological status, or geographic location (Clauw et al., 2024). While phenotypic variations can be directly observed, genetic variation is assessed using genotypic data. Population structure is the third major limitation as it plays a crucial role in GWAS. It determines the genetic relationships between individuals in a study group (Gowda et al., 2023). Understanding historical or genealogical connections among individuals is essential because uneven genetic relationships can impact the accuracy of results. If population structure is not accounted for, it may lead to misleading associations between genotype and phenotype (Altaf et al., 2025). One widely used software, STRUCTURE (version 2.3.4), helps analyze population structure by grouping individuals into subpopulations (Q-matrix) (Liu et al., 2025; Tian et al., 2025). Managing population structure is a significant challenge, as structured associations must often be adjusted to avoid bias. However, completely removing structured associations is not always the best solution, as it can affect the number of clusters and their proper assignment (Liu et al., 2025). Allele frequency distribution is another key factor influencing GWAS effectiveness is allele frequency distribution. In most populations, only a few genetic variants appear in a small number of individuals. If certain alleles are rare, detecting them becomes difficult unless they have a major effect on the trait being studied (Ahmed et al., 2024). Neglecting allele frequency during GWAS can result in inaccurate conclusions (Naveed et al., 2025). Most GWAS studies focus on common and rare variants, often considering allele frequencies greater than 5% (Mir et al., 2021). For instance, in a population of 500 individuals, an allele present in only 25 individuals is classified as rare, with a minor allele frequency (MAF) below 5%. Although rare alleles influence only a specific subset of the population, they may still be valuable for future breeding programs (Zhao N. et al., 2022). Linkage disequilibrium (LD) is also a major challenge that limits the power of GWAS. LD refers to the non-random association of alleles at different genetic loci within a population (Abdela, 2024). It is an important factor in GWAS, especially when identifying closely linked genetic markers (SNPs) that help pinpoint significant genomic regions (Clauw et al., 2024). If LD is not considered, both relevant and irrelevant alleles may be included in the analysis, leading to incorrect associations. LD analysis helps determine how many genetic markers are needed to cover the genome effectively (Peng et al., 2021). High LD values indicate that fewer markers are required. However, long-range LD can increase the likelihood of false associations, so calculating LD early in the analysis is necessary. LD values are typically measured using a disequilibrium matrix, which compares loci in pairs, using the most common statistical measures, D′ and *r* ^2^ (Shi et al., 2022).

### 4.4 Multi-omics approaches to mitigate heat stress

The physical appearance of plants, i.e., their phenotype in response to abiotic stresses, including heat stress, depends on the regulation of genes, mRNA, ions, metabolisms, and proteins. Recently, extensive use of omics approaches in elucidating plant tolerance mechanisms and stress response has helped to develop stress-resilient varieties. Despite the outstanding move forward in genomics (Yang et al., 2023; Sun et al., 2022; Purugganan and Jackson, 2021), there is still a need to explore the other omics approaches, such as ionomics, phenomics, metabolomics, proteomics, and transcriptomics to further improve our knowledge of genotype-to-environment and genotype-to-phenotype interaction. Before implementing any omics approach, it is essential to collect diverse plant germplasm, i.e., plant population comprised of both stress-susceptible and tolerant genotypes, to better understand the mechanisms associated with resistance.

#### 4.4.1 Transcriptomics

Transcriptomics, an important omics approach, used to understand and analyze gene expression at the transcription level, post transcriptional modifications, related transcripts and regulatory pathways (Corona-Gomez et al., 2022). Transcriptomics is also essential to explain how plants quickly reprogram transcriptional networks under stress, including heat stress (Satrio et al., 2024). NGS based transcriptomics appliances have laid the foundation for developing gene-specific molecular markers, identifying novel genes associated with heat resistance and facilitating marker-assisted breeding. For example, Masoomi-Aladizgeh et al. (2022) evaluated the effect of heat stress (>40°C) on different pollen development stages (from tetrads to mature pollen) in cotton. They exposed cotton plants to two different temperatures, i.e., 38°C during day and 28°C at night for five consecutive days and then subjected to transcriptomics analysis. According to their results, during pollen development, the molecular signatures were progressively downregulated under heat stress. This was more profound in leaves where most of the important protein’s abundance decreased significantly. At tetrads (early pollen development stage), genes activity upregulated and resulted in increased translation (support cell adaptation to heat stress) but significantly decreased as the pollen started developing towards maturity. Moreover, HSP were observed to be present in abundant quantity at tetrad stage but during lateral pollen stages and leaves their quantity reduced significantly. The study suggests that early pollen cells may prioritize processes that are not directly useful for heat tolerance, making them more vulnerable to stress. These molecular insights could help identify markers for breeding cotton varieties that are more resistant to heat, which is increasingly important in a warming climate. Similarly, Zhang et al. (2022) conducted transcriptomics analysis in cotton to identify the pollen specific genes associated with response to heat stress. In total, they identified 833 pollen DEGs, 1,066 anther and 1,111 pollen specific genes. They observed that both hormones and heat responsive *cis-regulatory elements* were abundant in the promoter region of anther specific genes indicating that these genes might be associated with response to heat stress. Moreover, only 10 DEGs out of 833 were found to be common with 1,111 pollen-specific genes, suggesting that pollen-related genes are only involved in pollen development rather than responding to heat stress. The promoter regions of these 10 genes were also found to be enriched in both MeJA and stress-related *cis-regulators*, confirming their involvement in both pollen development and heat stress responses. Besides cotton, transcriptomics has also been successfully implemented in other crops against heat stress. Hosseini et al. (2021) exposed different lentil genotypes to heat stress for a period of 4 h and based on transcriptomics analysis, identified around 4,327 DEGs (2,368 downregulated and 1959 upregulated). Downregulated genes were found to be associated with the ion’s transportation and membrane stability under heat stress. While upregulated genes were associated with the binding of microtubules, proteins and cell division as well as cell cycle. In another study, elevated night temperature in rice downregulated 695 DEGs associated with heat stress, protein folding and photosynthetic activities. On the other hand, 415 DEGs were upregulated associated with protein modifications, kaurene synthesis, RNA processing, carbohydrates metabolism and other signaling pathways. Liu et al. (2021) exposed a potato cultivar (Hezu088) to heat stress for a period of 8 h. The results showed that 160 DEGs associated with various cell activities such as HSPs, secondary metabolism, hormonal metabolism, protein and cell wall degradation as well as amino acid production. 538 DEGs were also downregulated associated with cytokinin metabolism, lipid metabolism, RNA regulation and signal transduction.

These findings from the above-mentioned studies illustrate the importance of transcriptomics in unraveling the plant responses to heat stress, identifying novel genes and useful insights into pathways associated with cotton and other plant’s adaptation to heat stress. Furthermore, transcriptomic studies have also contributed to understanding the underlying plant mechanisms, facilitating the introduction of climate smart heat resistance plant varieties in a short period.

#### 4.4.2 Proteomics

Proteomics is another omics approach that deals with the determination of protein components in plants and other organisms at a specific period of time and serves as an important link between transcriptomics and metabolomics (Niu et al., 2023). Proteomics analysis was introduced about 20 years ago (Hirano et al., 2004), but has recently been improved by the development of high-resolution and more accurate instruments (Aydoğan, 2024). These instruments have helped the breeders and plant scientist in understanding the plant response to changing environments at proteins levels (Raza et al., 2024). Heat stress is known to disrupts the proteins balance at cell level and ultimately regulates different mechanisms (Wu et al., 2021). For example, Khan et al. (2022) evaluated the effect of heat stress on cotton pollen development, i.e., pollen abortion by exposing two cotton cultivars HT-84021 (heat tolerant) and HT-H05 (heat sensitive) to various temperatures using 2-dimensional electrophoresis. According to their results, heat stress significantly disrupts the protein formation, translation, post translation modifications and discovered 307 DEPs spots in the anther of both HT-84021 and HT-H05. GO analysis further revealed that protein processing pathways in endoplasm play a crucial role in anther responses to elevated temperatures in combination with HSP. Among HSPs, BiP5 and HSP-70–17 were found to be involved in tolerance to heat stress and it was confirmed by Western blot gene expression analysis, respectively. Further, they observed that under heat stress the accumulation of ROS reduced significantly by the exogenous application of BiP5 and HSP-70–17 proteins. Thus, their finding suggests that both BiP5 and HSP-70–17 are the key cotton proteins in anther and can be helpful in developing high yielding and heat resistant cotton varieties. In another study, Zhang X. et al. (2021) investigated the effect of heat stress (>38°C) on insecticidal proteins concentration in a BT cotton cultivar Sikang-3 using label free quantitation proteomic technique. The concentration of insecticidal proteins significantly reduced, i.e., 65.2 ng/g under heat stress. The proteomic approach further revealed the downregulation and upregulation of 104 and 83 proteins, respectively. They also discovered 122 new proteins associated with heat stress response. Additionally, they further identified 14 KEGG pathways associated with protein synthesis. Out of 14, three KEEG pathways (endoplasmic protein processing, protein export and carbohydrates absorption and digestion) were more closely related to protein synthesis. In endoplasmic protein processing, through ubiquitin mediated proteolysis, plant ability to break down damage and misfold proteins increased. In protein export, the production of peptides was not significantly affected but their transportation to endoplasm reduced. In carbohydrates absorption and digestion pathway, plant ability to break down starch increased but had reduced efficiency to phosphorylate glucose, fructose and hexose. Proteomics analyzes have also been successfully implemented to other crops to discover novel proteins associated with resistance to heat stress and understand in details the proteomic based mechanisms linked to heat stress (Lee et al. 2007). For example, Castander-Olarieta et al. (2021) exposed *Pinus radiata* embryos to two different temperatures ranges, i.e., 60°C for 5 min and 40°C for 4 hours. According to their results, heat stress reduced initial embryonal masses around by 44% to 30.5%, while increasing somatic embryo production by 121.87–170.83 per Gram. Heat stress also caused long term alterations to proteins production machinery such as increased production of ribosomal proteins and decreased output of methionine-tRNA ligase enzymes. Proteomics analysis also displayed increase in proteins and fatty acids biosynthesis, proteins post transcriptional modifications, chaperones and HSPs, proteins transportation across cell membrane and carbohydrates. Heat stress also decreased enzymes associated with oxidative stress, nitrogen assimilation, glycolytic pathways and adenosylhomocysteinase protein. Similarly, another study conducted by Wang et al. (2021) discovered 1,591 DAPs associated with metabolites and carbohydrates transport, energy conversion and production, catalytic activity, molecular transporter and structural activities in two Capsicum cultivars (heat sensitive 05S180 and heat-tolerant 17CL30) under elevated temperature. It has been reported that in wheat under heat stress, DAPs are involved in reduced glycolysis and photosynthesis but more gliadins and translation (Chunduri et al., 2021). Moreover, *Brachypodium distachyon* proteomics analysis discovered 46 DAPs (42 downregulated and four upregulated) associated with lower protease activities, lignification and expansion of cell wall (Pinski et al., 2021).

Proteomic studies have uncovered how plants respond and adapt to temperature stress by altering proteins involved in photosynthesis and defense. Identifying these key proteins provides valuable insights for breeding temperature-resilient crops.

#### 4.4.3 Ionomics

Ionomics helps in determining how plants accumulate, absorb and distributes nutrients from soil to cells under different environmental conditions including heat stress (Xiao et al., 2021). The word ionome refers to the sum of inorganic essential nutrient that are necessary for plant adaptation and different cell mechanisms in small amount. Techniques linked to ionomics, involves elements composition profiling and deviations under different stresses (Singh et al., 2022). Ionomics is mostly used to study plant response to drought, salinity and metal stress, relatively few studies other than cotton have explored the application of omics in understanding plant responses to heat stress. A study was conducted to profiled the ionomic of a mutant and wild-type tobacco plants subjected to heat stress. The results revealed a significant decrease in Zn and Fe concentrations and an increase in Mg and Ca. The concentrations of these ions also greatly varied in roots and ariel parts of plants, suggesting the alterations in ions uptake due to heat stress (Ardini et al., 2016). Another study conducted to elucidate ionomics analysis of quinoa seeds exposed to different regimes of heat stress. Prolonged exposure to heat stress significantly alters the nutrient composition, quality, size and development of the seed (Tovar et al., 2022). These studies offer valuable insights in understanding the role of ions in heat stress. However, these studies can be used as an example or footprint in cotton to identify the role of ions related to heat stress.

#### 4.4.4 Metabolomics

**FIGURE 2:**
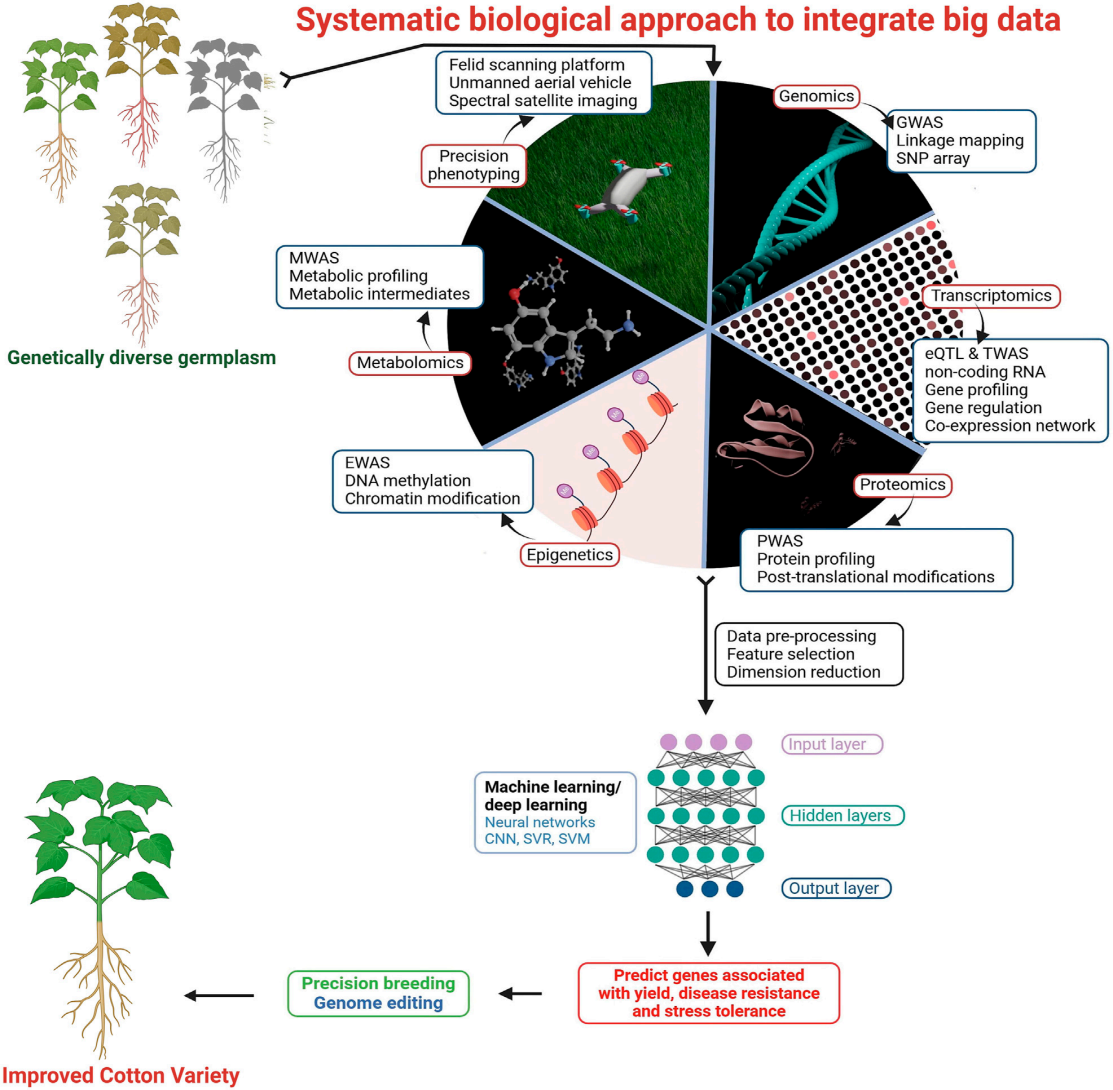
A systematic biological approach is utilized to integrate large-scale data for the identification and characterization of plant cellular molecular mechanisms and pathways. Advancements in high-throughput technologies across various omics fields including phenomics, metabolomics, proteomics, transcriptomics, epigenomics and genomics have significantly enhanced the exploration of plant genetic resources, aiding in the discovery of novel alleles. Multi-omics datasets undergo preprocessing steps such as data cleaning, feature selection, and dimensionality reduction to refine the information. Deep learning and machine learning methodologies systematically integrate these multi-omics layers, offering a comprehensive understanding of the pathways and molecular mechanisms that contribute to complex agricultural traits. Note: this figure was generated using BioRender.

Metabolomics is another omics approach equipped with modern computational biology tools, that provides detailed analysis of hormones and metabolites (both primary and secondary) produced by an organism or cells during biological process (Sprink et al., 2015). Metabolomics helps uncover how plants interact with their environment, particularly under changing climatic conditions like elevated temperatures Figure 2. Certain metabolites have the ability to specify mechanisms associated with stress adaptation by modulating physiological responses, growth patterns and signaling cascade networks (Niu et al., 2023; Gupta et al., 2014). Recently, researchers are focusing on identifying stress associated pathways and metabolites in cotton as well as in other plants. For example, Melandri et al. (2021) evaluated the effect of heat stress on the metabolomics of 22 cotton genotypes grown continuously for 2 years (2018 and 2019). Results revealed significant negative impact of heat stress on the metabolome of almost all genotypes across both growing seasons, where reduced lint length, quality, quantity, leaf area and plant height. In 2018, a total of 307 metabolites, 217 were identified to be significantly affected (approximately 70.7%). While in 2019, a total of 521 metabolites, 451 were identified to be significantly affected (approximately 86.6%). However, few genotypes displayed a great tolerance to heat stress by altering membrane lipid chemical composition. In another comparative analysis study, the effect of heat stress on fiber metabolome was analyzed. Heat stress significantly decreased fiber quality by reducing sugar phosphate, sugar acids, sugar alcohols and free sugars. Moreover, metabolomic process linked with cytoskeleton, cell wall, and biosynthesis of carbohydrates were also suppressed (Naoumkina et al., 2013). Besides cotton, metabolomics has also been successfully applied to other crop against heat stress. For example, to understand the effect of heat stress on metabolomics in cucumbers and tomato. The results from each study revealed that know-down of *HsfB1* gene in tomato under stress accumulated putrescine, sucrose and glucose which are involved in response to heat stress. While overexpression of *HsfB1* produced substances from the phenylpropanoid and flavonoid pathways, as well as some caffeoyl quinic acid compounds, which improved heat tolerance (Paupière et al., 2020). In the other study, when cucumber plants exposed to high temperature (30°C at night and 38°C during the day for consecutive 12 h), identified in total 125 metabolites (26 downregulated and 99 upregulated) that changed under heat stress. These changes occurred in four key pathways, first, threonine, serine, and glycine metabolism, second, chlorophyll and porphyrin metabolism, third, nucleotide and amino sugar metabolism, and fourth, plant hormone signaling (Chen et al., 2023b).

Metabolomics studies have provided valuable insights into the complex responses of plants to heat stress, uncovering adaptive strategies and dynamic metabolic pathways. The identification of key markers enhances our understanding of stress adaptation and tolerance mechanisms across various plant species. These findings have deepened our knowledge of plant × environment interactions and contribute to the development of temperature-resilient crop plants in a short period of time.

#### 4.4.5 Phenomics

Phenomics also called phenotype, is the study of physical appearance of plant, has gained notable importance in the post-genomic era to understand plant responses to environmental stressors, such as heat stress (Singh et al., 2018). Phenomics analysis requires highly accurate phenotypic data on various plant’s traits, collected through advanced high through phenotyping tools and complemented by genomic insights. Analyzing phenomic traits can reveal mechanisms that are associated with genotypes to specific phenotypes (Houle et al., 2010). Various techniques are employed to study plant phenomes, including integrated imaging, spectroscopy, fluorescence, thermal imaging, infrared and visible light approaches (Singh et al., 2018). Numerous studies have effectively utilized phenomics to investigate cotton and other plants responses to heat stress. For example, Wu et al. (2014) evaluated the effect of heat stress (45°C for 24 h) on 44 wild-types of cotton using phenomics technique called Chlorophyll Fluorescence Imaging (CFI). The results revealed alterations in photosynthetic rates (especially in photosystem II) by reducing total chlorophyl contents significantly under heat stress. CFI helped in selecting tolerant lines based on PSII performance. In another study, Pauli et al. (2016) applied a high-throughput phenotyping system across the cotton field and equipped it with a group of sensors to collect phenotypic data for traits like plant height, reflectance, leaf area, vegetative index and canopy temperature. According to their results, leaf area, vegetative index and canopy temperature showed moderate-to-high heritabilities, suggesting that these traits were reliable and consistent for measuring genetic differences among cotton varieties. Moreover, for traits like vegetative index and canopy temperature, QTL expression changed at different growth stages of the plant. This suggest that certain genes played a stronger role at specific times.

Phenomics can still be considered in its early stages, especially when it comes to understanding how heat stress affects crops growth and development. However, it shows great potential and can be very useful in accelerating crop breeding programs to improve heat stress tolerance (Basavaraj and Rane, 2020). Combining high throughput genomics (i.e., NGS technologies) with phenomics (phenotype) can offer valuable insights and help in developing climate-smart crops (Marsh et al., 2021). By using bioinformatics tools to integrate phenotypic and genotypic data, scientists can build detailed and huge datasets for each plant species or groups specifically. This can defiantly make it easier to identify and select plant traits that help crops adapt to climate challenges (Marsh et al., 2021). Although combining different omics strategies as well as approaches is highly beneficial but it might be a challenging task to do. New technologies, like rapid genomic advancements, high-throughput phenomics, and tools that analyze environmental relationships, are essential for improving traditional breeding methods and increasing genetic progress (Crossa et al., 2021).

#### 4.4.6 Integration of multi-omics approaches

Multi-omics data integration is a valuable strategy combining information from various high-throughput omics technologies, including ionomics, metabolomics, proteomics, transcriptomics and genomics to comprehensively understand the complex biological systems. This approach has been extensively applied in different crops, including cotton, to explore the molecular mechanisms involved in abiotic stress tolerance, such as heat stress (Singh S. et al., 2024; Fang et al., 2023; Lu et al., 2025). Understanding these mechanisms is essential for developing stress-resistant crop varieties. By integrating multi-omics data, researchers can obtain a more complete picture of how plants respond to heat stress at the molecular level (Han et al., 2022b). The process of integrating multi-omics data to study crop stress tolerance follows several key steps. The first step, experimental design, involves planning and structuring experiments to expose crops to specific abiotic stress conditions while including appropriate control groups for comparison (Fan et al., 2025). This step ensures proper sample collection across different omics platforms, including metabolites, proteins, RNA and DNA. The second step, data generation, uses high-throughput omics technologies such as metabolomics to profile metabolism, RNA-seq for transcriptomics, proteomics through mass spectrometry and whole genome sequencing for genomics (Roychowdhury et al., 2023). These methods generate large-scale datasets for each omics layer. In the data pre-processing step, preprocessing procedures and quality control specific to each omics dataset are applied. This includes metabolomic and proteomic data missing values imputation and normalization, quality control, and transcriptomic and genomic data alignment and read trimming (Ahmed et al., 2024). If multiple experiments are conducted, batch effects are also removed to ensure data consistency. The final step, data integration, involves computational techniques to merge multi-omics datasets. This step requires robust statistical methods to achieve meaningful integration of different omics layers (Naveed e al., 2025). Functional and statistical networks are employed to analyze the interconnections between datasets, allowing for better validation and visualization of results obtained through multi-omics approaches. For example, Wang et al. (2015), conducted a proteomic analysis using iTRAQ on ovules of upland cotton and its fuzzless-lintless mutant under heat stress, identifying 2,729 proteins that accumulated predominantly at anthesis in wild-type ovules. RNA sequencing (transcriptomics) further confirmed that 2,005 of these proteins were also upregulated at the transcript level. Proteins associated with lipid metabolism, hormone regulation, small-molecule metabolic processes abd carboxylic acid metabolism showed significantly higher expression in wild-type ovules. qrt-PCR validated the increased expression of 26 genes involved in these pathways. Among these, *GhPAS2* catalyzes the third step in VLCFA biosynthesis, was highly accumulated in wild-type ovules at anthesis. Heterologous expression of *GhPAS2* restored viability in a *Saccharomyces cerevisiae* haploid strain lacking PSH1, which is essential for PAS2 activity. Furthermore, the application of acetochlor (ACE), a VLCFA biosynthesis inhibitor, along with gibberellic acid, significantly inhibited fiber cell initiation in unfertilized cotton ovules. This study provides new insights into cotton fiber cell development under heat stress by integrating transcriptomic and proteomic data. Similarly, Han et al. (2022a), conducted integrated multi-omics analysis to understand the molecular mechanisms of cotton’s response to both drought and heat during the boll and flowering stages, metabolomic and transcriptomic analyses were conducted on two introgression lines: T307 and S48. The results revealed that drought-sensitive and drought and heat tolerant lines activated broad-spectrum drought responses, including MAPK signaling pathway, ABA signaling and amino acid synthesis. However, the genotype T307 exhibited additional responses due to its imported gene fragments and missing sequences, leading to enhanced endoplasmic reticulum protein processing, improved photosynthetic pathways in leaves, and increased membrane solute transport in roots. These mechanisms were either absent or not activated in the drought-sensitive line S48, explaining their differences in drought resistance. A virus-induced gene silencing (VIGS) assay of differentially expressed ATP-binding cassette transporter genes (particularly in roots) and HSP genes (primarily in leaves) confirmed their significant roles in drought and heat tolerance. The combined metabolomic and transcriptomic analysis highlighted the importance of ER stress-related root-specific ABC transporter genes and HSP genes in cotton’s adaptation to drought and heat stress. These findings offer new insights into the molecular basis of drought and heat resistance in cotton. Wang Z. et al. (2023), conducted integrated multi-omics approaches in colored cotton also known as eco-cotton, produces naturally pigmented fibers but has lower yield and quality compared to white cotton. The regulatory genes controlling pigment synthesis and fiber quality are not well understood. They analyzed proteomic and transcriptomic changes during fiber development a white cotton cultivar and in a brown cotton cultivar (Z161) to identify key molecular mechanisms. Differentially expressed genes and proteins showed a significant positive correlation in their expression trends. Enrichment analysis revealed that Z161 exhibited upregulation of genes involved in glutathione metabolism, phenylalanine metabolism, flavonoid biosynthesis and fiber elongation. Additionally, 15 MYB-bHLH-WD40 complex genes, 164 glycosyltransferase genes and transcription factors such as NAC (7), ERF (11) and C2H2 (12) and were preferentially expressed in Z161. Weighted correlation network analysis highlighted energy metabolism and fatty acid synthesis as key pathways influencing fiber development in both cultivars. The identification of 15 hub genes provides valuable insights for improving balancing pigment and fiber quality in brown cotton through genetic modification. Besides heat stress, integrated multi-omics approaches have also been applied to studying salt stress in cotton. For example, Ju et al. (2023), examined how cotton roots respond to salt stress using metabolome and transcriptome analyses, supported by physiological measurements. Cotton roots were treated with three salt and potassium supplementation conditions. The findings revealed that ROS scavenging pathways, hormone metabolism and ion transport play essential roles in cotton root adaptation to salt stress. Salt stress led to ion toxicity and oxidative damage by disrupting hormone balance and reducing the expression of antioxidant and potassium transporter genes. However, potassium supplementation helped mitigate salt stress damage by maintaining hormone homeostasis and ion and enhancing ROS removal. They identified key metabolites, regulatory genes and biological pathways involved in potassium mediated salt stress adaptation. It constructed a gene metabolite interaction network, providing new insights into how potassium helps cotton and other crops cope with salt stress. These findings contribute to cotton genetic improvement and optimized cultivation strategies. Peng et al. (2018), combined transcriptomics and proteomic data to identify genes that show differential expression at both the mRNA and protein levels. However, for most highly differentially abundant proteins, no significant changes were observed in their corresponding mRNA levels. This discrepancy may be due to global shifts in alternative splicing and miRNA activity under salt stress. Their findings suggest that certain salt stress-responsive proteins can influence miRNA levels and regulate alternative splicing in upland cotton. A detailed comparison between salt-sensitive and salt-tolerant genotypes identified 85 and 63 candidate proteins/genes linked to salt tolerance, respectively. Further analysis predicted an interaction network of 158 proteins/genes, revealing two key clusters centered around cytochrome oxidase and ATP synthase in mitochondria. These results highlight the critical role of mitochondria in energy metabolism and the production of resistance related proteins during salt stress adaptation.

Moreover, various strategies can be utilized for data integration, including ML algorithms, network and correlation-based approaches (Roychowdhury et al., 2023). PaintOmics 4, a web-based platform, facilitates the integration of multi-omics datasets by mapping them onto biological pathways (Liu et al., 2023). Effective data integration is essential for combining information from diverse sources to develop models capable of predicting complex traits and improving prediction accuracy. To enhance phenotype prediction, a range of statistical models including both nonlinear and linear have been developed and are widely applied. Linear models such as BSLMM, LMMs, GBLUP and PLMM with Generalized Method of Moments Estimator are commonly used for modeling multi-omics data with high predictive accuracy (Ahmed et al., 2024). On the other hand, ML techniques, which encompass unsupervised and supervised learning approaches, utilize statistical inference to analyze large and complex datasets. In supervised learning, the primary goals are regression and classification, while unsupervised learning is often employed for dimensionality reduction (DR), association and clustering (Roychowdhury et al., 2023). DR is particularly useful in high-dimensional biological data, as it reduces the number of variables considered, aiding in data interpretation. These methodologies help uncover relationships and interactions among molecules across various omics layers (Fuck et al., 2024). Functional analysis plays a key role in interpreting integrated multi-omics data by identifying molecular mechanisms underlying abiotic stress responses. This includes functional annotation of crucial metabolites, proteins and genes, pathway enrichment and Gene Ontology analysis (Khan et al., 2023a). Network analysis, on the other hand, constructs biological interaction networks, such as protein to protein interactions networks and co-expression to pinpoint key regulatory genes or proteins involved in stress responses (Kan et al., 2023). Finally, experimental validation is crucial for confirming the biological significance of metabolites, proteins and candidate genes identified through integrated analyses. Techniques such as targeted metabolomics, Western blotting and qPCR can be used to validate these findings and elucidate post-translational and post transcriptional mechanisms regulating gene expression (Khan et al., 2022). Experimental verification is a critical step in ensuring the reliability of data and uncovering regulatory mechanisms in stress adaptation.

## 5 Conclusion

Heat stress is one of the prime factors limiting the yield of cotton around the world and need to be addressed. It causes membrane leakage, production of ROS, nutritional imbalances and leads to water logging condition and unavailability of water and oxygen to roots and all these changes collectively have adverse effect on the growth and development of crop. It has been reported that every 1°C increase in temperature leads to a 10% decrease of cotton yield. Due to continuous rise in temperature, cotton crop is facing serious issues in growth and development. Most of the cotton genotypes are sensitive to elevated temperature and do not perform well under heat stress in field. As cotton is known as heat loving plant, still its response to heat stress at different developmental stages is diverse.

An efficient approach to face heat stress is the production of heat tolerant varieties. However molecular mechanisms of heat tolerance have been explored in many crops and this knowledge could be utilized to breed for heat tolerant cultivars in cotton. In cotton, abiotic stresses are seen to be interrelated, such as heat stress being accompanied by drought stress, and these stresses are thought to share some genetic elements. Heat stress severely affects the cotton crop’s biochemical, physiological, and molecular processes, which ultimately lead to poor growth of the crop, accompanied by low photosynthetic rate and yield.

These biochemical, molecular, and physiological parameters could be used to screen for heat tolerant genotypes under field and glasshouse conditions. As yield has negative correlation with abiotic stress tolerance, linkage drag has proved to be fatal for improvement of cotton germplasm for heat tolerance by direct selection. However molecular markers played revolutionary part in the identification of desired QTLs in diverse populations and proved extraordinarily helpful in understanding the genetic base of heat tolerance. The development of mapping technologies and discovery of molecular markers has enabled the scientists to transfer the desired genes from one genotype to the other by the MAS (molecular assisted breeding) process.

By using wild genetic resources in MAS, the linkage drag could be avoided. With the development of this field and due to its efficiency, scientists started doing work in this domain, which led to the discovery of many desired QTLs in diverse crop species for heat tolerance. However, a little work has been done to identify the genes for heat tolerance, research is still in process to identify more genes with the help of transgenic techniques. Through the use of genetic engineering, many genes associated with heat tolerance have been inserted in cotton to improve genetic resource/germplasm for heat tolerance. However, these identified genes are not used commercially in breeding programs for the improvement of cotton. Due to the complex nature of heat response and interconnection with other processes of development and growth, unfavorable impacts of genetic transformation have been reported.

The present status of transformed cotton with increased heat tolerance is not according to the demand. This gap between demand and requirements is the result of less attention paid by scientists to this topic in the past. Future research should be designed with the main focus on the usage of heat tolerant genes on maximum, and the production of more heat tolerant materials. It is now projected that a more diverse population will be developed with a combination of parent genomes with more resolving QTL mapping and intensive phenotyping for multiple abiotic stresses with the help of single nucleotide markers (SNPs).

The QTLs suspected to have more heat tolerance will be explored through marker assisted selection (MAS) and heat-tolerant genes will be introduced into the high yielding genotypes of cotton. Moreover, to increase the efficacy of developing heat tolerant materials rapid, accurate, and high throughput procedures should be developed to screen the available germplasm for multiple stresses at the same time.

Genome editing, molecular breeding and next-generation sequencing tools including CRISPR-Cas and multi omics approaches have also laid the foundation for developing climate resilient crop varieties. CRISPR has revolutionized the field of genetics by introducing and removing the wanted and unwanted genes with plant species making them tolerant to adverse climatic conditions. Advancements in sequencing technologies have let scientists to study the underlying plant genetics in more detail and find the causal genetic variants that are responsible or associated with responses to heat stress. The new era of GWAS, PWAS and TWAS have made the development of new crop varieties easier in a very short period of time just by dealing with a single SNP rather than a whole gene. The combination of multi omics approaches such as genomics with high throughput phenotyping and also with genome editing in the future can be very helpful in speeding up the breeding programs and developing climate smart varieties.
